# Quantitative evaluation and optimization of long-term care insurance policy texts in pilot cities of Guangxi Zhuang autonomous region

**DOI:** 10.3389/fpubh.2026.1853798

**Published:** 2026-07-24

**Authors:** Shuang Liang, Ling Li, Jing Luo, Lang Jiang, Zong-Fan Yang

**Affiliations:** School of Public Health and Management, Guangxi University of Chinese Medicine, Nanning, Guangxi, China

**Keywords:** content analysis, long-term care insurance, PMC index model, policy instrument theory, stakeholders

## Abstract

**Objective:**

To systematically evaluate the quality and internal consistency of the long-term care insurance (LTCI) policy documents in five pilot cities of Guangxi Zhuang Autonomous Region, explore the allocation structure of policy instruments and the interaction pattern of stakeholders, and identify the strengths and weaknesses of the policy system.

**Methods:**

A total of nine core policy documents from five cities including Hezhou, Nanning, Liuzhou, Beihai and Guigang were collected. The PMC index model and the policy tool analysis framework were adopted, and an evaluation index system was established after text mining with ROSTCM software. Two researchers conducted independent coding, and consensus negotiation was adopted to guarantee coding reliability (Holsti coefficient *R* = 0.9866, Kappa value of PMC scoring = 0.918).

**Results:**

The average PMC index of policies in the five Guangxi cities was 6.59 (good grade), with Hezhou (7.23) and Beihai (7.07) rated excellent. Environmental policy instruments occupied a dominant position, accounting for 41.47%, while supply-side instruments (25.08%) and demand-side instruments (33.44%) were relatively insufficient. Specifically, facility construction (6 items) and human resource development (16 items) were severely inadequate, and payment provisions (127 items) made up 63.50% of all demand-side instruments. Stakeholders presented the feature of “administrative dominance with insufficient diversified participation”: administrative departments accounted for 40.30%, social donors only accounted for 1.00%, and beneficiaries merely took up 1.33% in supply-side policy instruments. Policy timeliness (X2 = 0.40) and incentive and restriction mechanisms (X9 = 0.56) were the common deficiencies among the five cities.

**Conclusion:**

The LTCI policy in Guangxi has initially formed an institutional framework suited to ethnic minority areas. It has met the national framework requirement of “ensuring basic needs with wide coverage” in terms of the coverage of insured parties and adaptation to relevant fields. However, policy timeliness and incentive and restraint mechanisms remain prominent shortcomings, and an effective connection has not yet been established between the construction of the institutional framework and its long-term operation. Improvements should be made in four aspects: promoting the county-level distribution of nursing facilities and professionals, optimizing the structure of demand-oriented policy instruments, institutionalizing the combination of progressive coverage and consumption vouchers, and filling gaps in full-chain supervision.

## 1 Introduction

Population aging and societal aging are severe challenges facing contemporary Chinese society, accompanied by a continuous increase in the number of disabled and semi-disabled older adults (1) which has generated substantial demand for professional and regular long-term care services. To address this systemic risk, China officially initiated the pilot program for the long-term care insurance (LTCI) system in 2016 (2) and expanded the pilot scope in 2020 (3), aiming to establish a social insurance-based LTCI system through a risk-pooling mechanism (4) to safeguard the basic care needs of disabled individuals.

Compared with early pilot regions, late-adopting regions such as Guangxi not only undertake the universal institutional goals of “basic protection and broad coverage” but also face the unique context of unbalanced economic and social development and a relatively weak foundations in care services in ethnic minority areas (5). With care service supply capacity falling below the national average, and notable gaps in economic development, demographic structure, and fiscal capacity relative to first-round pilot cities, such disparities are likely to give rise to distinct policy logic: fiscal constraints may make policies more reliant on administrative regulation than financial investment; the urban–rural dual structure in ethnic regions may require a progressive coverage pathway; and weak care service foundations may push the policy focus toward establishing institutional rules rather than directly expanding service capacity. However, existing research has paid insufficient attention to policy texts in such “late-pilot ethnic minority regions.” At the national level, Sun et al. (6) conducted a quantitative evaluation based on the PMC index model; Xue et al. (7) carried out a policy assessment from the perspective of common prosperity; Xiao et al. (8) and Peng et al. (9) applied the PMC index for policy evaluation; Li and Jiang (10) used a policy instrument framework; and Zhou et al. (11) analyzed policy instruments in national pilot cities. At the local level, Jiang (12) examined institutional optimization in Henan Province, and Huang (13) investigated satisfaction with the pilot program in Tianjin. The analytical scope of these studies has largely focused on the national level or on early pilot regions, primarily summarizing experiences or examining macro-level institutional design. Consequently, systematic quantitative evidence is still lacking regarding the structural characteristics and quality of policy design in late-pilot areas—particularly in multi-ethnic regions—under the overarching national framework. Therefore, it is necessary to conduct a targeted quantitative evaluation of LTCI policies in Guangxi and other similar late-pilot areas to reveal the structural features and potential weaknesses of their policy design.

Accordingly, this study focused on five pilot cities in the Guangxi Zhuang Autonomous Region. By collecting core policy documents and introducing the Policy Modeling Consistency (PMC) index model and policy instrument analytical framework, this study aimed to: (1) quantitatively evaluate the overall quality and internal consistency of Guangxi’s LTCI policies; (2) deeply analyze the deployment structure of policy instruments and interaction characteristics of stakeholders; (3) identify strengths and weaknesses of the current policy system, and propose targeted optimization suggestions. This study not only helps objectively assess the preliminary effectiveness of the “Guangxi Model” but also provides a reference for other late-adopting pilot regions and ethnic minority regions to improve their LTCI system.

## 2 Materials and methods

### 2.1 Data sources

As of January 2026, LTCI pilots had been implemented in Hezhou, Nanning, Liuzhou, Beihai, and Guigang in the Guangxi Zhuang Autonomous Region. A total of 13 policy documents related LTCI were retrieved from official websites of the people’s governments and healthcare security bureaus at the autonomous regional and municipal levels, as well as legal databases such as Pkulaw. To ensure consistency and comparability of analytical objects, the following screening criteria were applied: (1) select iconic and comprehensive main policy documents and core implementation rules of each pilot city; (2) exclude procedural documents such as circulars and approvals and supporting operational guidelines; (3) ensure equivalent administrative effectiveness levels of the final included texts. Ultimately, nine core policy documents from each city were included (Table 1).

**Table 1:** List of LTCI Policy Documents in Guangxi.

Policy number	Policy name	Document number	Issuing authority
P1	Notice on Printing and Distributing the Pilot Work Plan for the Long-term Care Insurance System of Hezhou City	HeZheng Gui [2018] No. 8	Hezhou Municipal People’s Government
	Notice on Printing and Distributing the Detailed Implementation Rules for LTCI in Hezhou (Trial)	He Renshe Fa [2018] No. 93	Hezhou Municipal Bureau of Human Resources and Social Security, Finance Bureau
P2	Implementation Opinions on Promoting the LTCI System in Nanning	Nanfugui [2023] No. 26	Nanning Municipal People’s Government
	Notice on Printing and Distributing the Detailed Implementation Rules for LTCI in Nanning	Nan Yibaogui [2024] No. 2	Nanning Municipal Healthcare Security Bureau, etc.
P3	Implementation Opinions on Establishing the LTCI System in Beihai	Beizheng Fa [2024]	Beihai Municipal People’s Government
	Notice on Printing and Distributing the Detailed Implementation Rules for LTCI in Beihai	Bei Yibao Fa [2024] No. 15	Beihai Municipal Healthcare Security Bureau, etc.
P4	Notice on Printing and Distributing the Implementation Opinions on Establishing the LTCI System in Liuzhou	Liu Yibaogui [2025] No. 1	Liuzhou Municipal Healthcare Security Bureau
	Notice on Printing and Distributing the Detailed Implementation Rules for LTCI in Liuzhou	Liu Yibaogui [2025] No. 2	Liuzhou Municipal Healthcare Security Bureau
P5	Notice on Printing and Distributing the Implementation Plan for Establishing the LTCI System in Guigang	Guizheng Bangui [2025] No. 9	Guigang Municipal People’s Government

It should be noted that the number of included policy documents varies across cities (2 documents each for P1, P2, P3 and P4, and 1 document for P5). Differences in document length and level of detail may affect the absolute frequency of policy tool coding for each city. Therefore, in the cross-city comparison of this study, percentage analysis is adopted alongside absolute frequency to reduce bias caused by variations in document length.

### 2.2 Research tools and methods

#### 2.2.1 Framework construction and reliability assurance

1. Analytical framework development

The analytical framework of this study is grounded in two theoretical foundations. The first is Policy Instrument Theory. Rothwell and Zegveld (14) classified policy instruments into three categories: supply-oriented, demand-oriented, and environment-oriented. Supply-oriented instruments emphasize direct government promotion of policy goals through resource inputs (funding, facilities, workforce, and standards); demand-oriented instruments focus on stimulating market demand through public procurement, service outsourcing, and consumption subsidies; and environment-oriented instruments aim to shape the institutional environment through regulatory control, strategic planning, and collaborative oversight. Drawing on this classification logic, and adapting it to the specific content of LTCI policies, this study categorizes 12 specific policy instruments into the above three types.

The second theoretical foundation is Stakeholder Theory. Freeman (1984) defined stakeholders as “any group or individual who can affect or is affected by the achievement of the organization’s objectives.” In the context of LTCI policy, multiple stakeholders—including government agencies, insured individuals, care service providers, and commercial insurance institutions—are interconnected through various interests and interactions. The design and implementation effectiveness of policy instruments depend substantially on the degree of stakeholder participation and the quality of their interactions. Based on these two theoretical pillars, this study identifies eight categories of LTCI stakeholders and constructs a two-dimensional “policy instrument–stakeholder” analytical framework.

1. Coding reliability test

This study employed content analysis to code the nine selected LTCI policy texts. Operative provisions and measures within the policy documents were treated as coding units, extracted and organized according to the format of “policy number–specific provision.” The unstructured textual data were systematically compiled into Excel spreadsheets to generate quantifiable analytical data. Through this process, a total of 1,143 coding analysis units were identified. These units were then manually coded, classified, and summarized based on the analytical framework, producing a policy content analysis coding table structured as “policy number–specific provision–policy instrument–stakeholder.”

To ensure the reliability of the coding and classification process, two coders jointly performed the coding and statistical analysis of policy analysis units. Both coders thoroughly reviewed the coding manual and strictly adhered to the established coding protocols. Following the coding framework and operational requirements, they independently coded the organized quantitative data. Upon completion, the total number of coding units identified by each coder and the number of units on which both coders agreed were tallied. Inter-coder reliability was assessed using Holsti’s coefficient, calculated according to the following formulas:


$$K=\frac{2\times M}{N_{1}+N_{2}}\;\;\;$$ (1)

$$R=\frac{n\times K}{1+K(n-1)}\;\;$$ (2)

Where N₁ and N₂ represent the total number of coding analysis units identified by each of the two coders, respectively; M denotes the number of coding units on which both coders reached agreement; K represents the inter-coder agreement coefficient using Equation 1; n indicates the number of coders involved; and R is the coding reliability coefficient using Equation 2. The statistical results showed that N₁ = 778, N₂ = 773, and M = 738, yielding K = 0.9736 and R = 0.9866, using Excel. The inter-coder agreement rate exceeded 80%, which is sufficient to support subsequent analyses.

In addition, for the assignment of scores to the secondary variables of the PMC index model, two coders independently rated 200 PMC secondary variable items (5 cities × 40 secondary variables). The Kappa coefficient calculated using SPSS was 0.918 (*p* < 0.001), indicating excellent inter-coder agreement. The complete coding manual is available in the Supplementary material.

#### 2.2.2 PMC index model evaluation methodology

The Policy Modeling Consistency (PMC) index model, proposed by Mario Arturo Ruiz Estrada (15, 16), is grounded in the “Omnia Mobilis” assumption—the premise that all relevant variables should be taken into account in policy evaluation, rather than assuming “all other things being equal” (*Ceteris Paribus*) as in traditional economic approaches. Based on this premise, the PMC model adheres to a principle of comprehensiveness, incorporating as many potentially relevant variables as possible and assigning equal weights to each variable to mitigate the subjective preferences of the evaluator (15). This study adopts the PMC methodological framework established by Estrada (2011), draws on the variable configuration schemes applied in Chinese LTCI policy evaluations by Zhang et al. (17) and Chen (18), and adapts these to the actual content of the policy texts from the five Guangxi cities to construct a PMC evaluation model tailored to Guangxi’s LTCI policies. The main procedures include variable classification and parameter identification, construction of a multi-input–output table, calculation of the PMC index, and generation of PMC surface diagrams.

1. Variable classification and parameter identification

**Figure 1:**
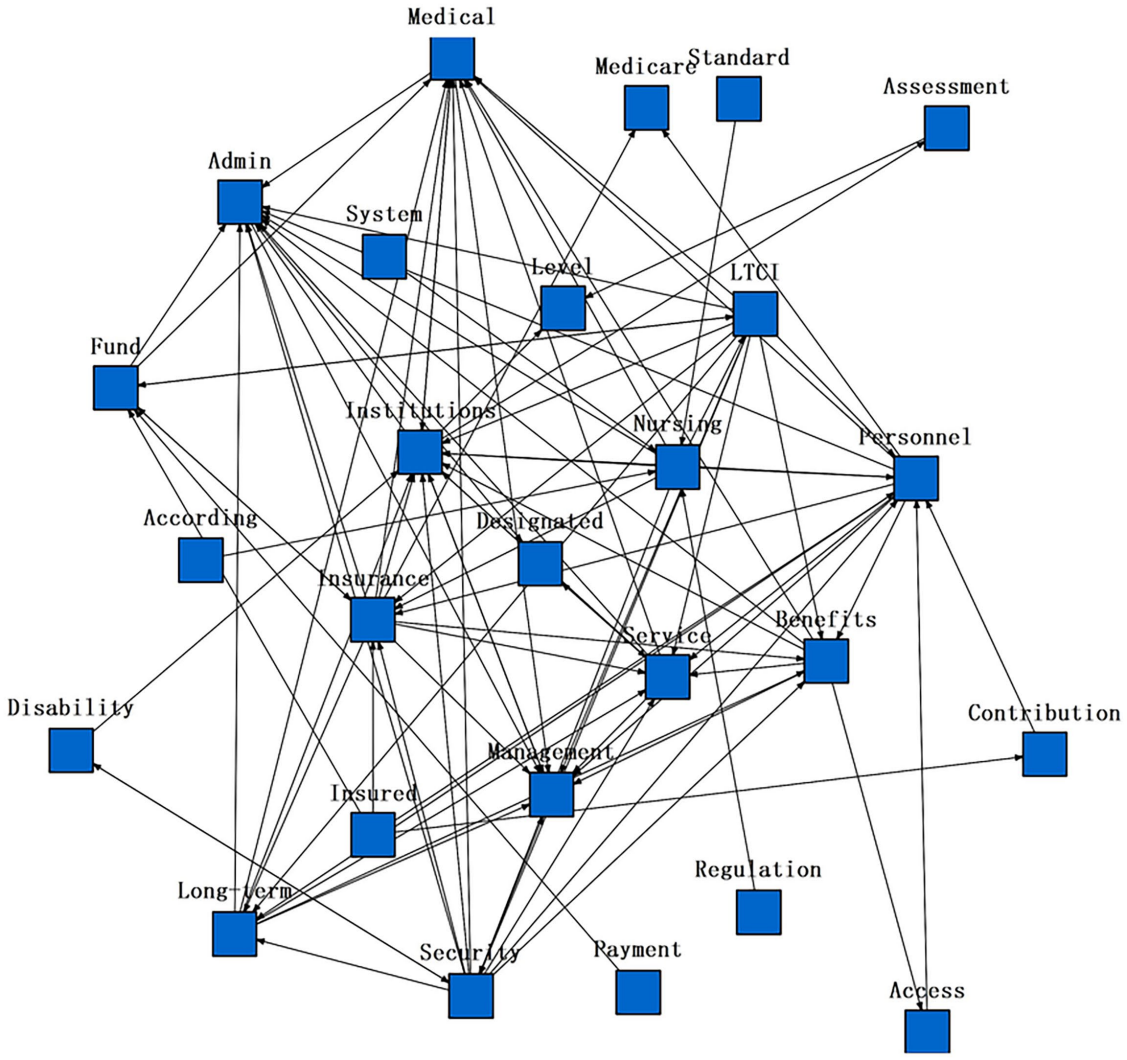
Social network semantic map of LTCI policy texts in Guangxi.

This study used ROSTCM software to perform word segmentation and word frequency analysis on the collected and screened policy texts. After removing noise words such as “work,” “basic,” and “implementation,” the top 60 high-frequency words across the five policy texts were extracted (see Table 2), and a social network semantic map was generated (see Figure 1). The high-frequency word analysis revealed that, centered around the core concept of LTCI, terms related to “services,” “management,” “protection,” “benefits,” “institutions,” and “funds” appeared frequently.

**Table 2:** Summary of high-frequency words in LTCI policy texts.

Serial number	Vocabulary	Word frequency	Serial number	Vocabulary	Word frequency	Serial number	Vocabulary	Word frequency
1	Nursing	682	21	Regulation	115	41	Policy	49
2	Service	513	22	Employee	110	42	Formulate	48
3	Institution	482	23	Department	101	43	Entrust	47
4	LTCI	408	24	System	99	44	Monthly	47
5	Insurance	373	25	Medical insurance	91	45	Home-based	47
6	personnel	319	26	Evaluation	89	46	Ability	44
7	Long-term	307	27	According to	84	47	Country	44
8	Medical	284	28	Level	84	48	Base on	43
9	Disability	268	29	Cost	81	49	Undertake	41
10	Management	253	30	Establish	72	50	Project	41
11	Handled	238	31	Society	65	51	Premium	40
12	Safeguard	228	32	Severe	62	52	Coordinate	40
13	Compensation	219	33	Unit	62	53	Funding	39
14	Assessment	204	34	Settlement	58	54	cooperation	39
15	Fund	187	35	Protocol	58	55	Pension	39
16	Insurance participation	170	36	Condition	56	56	Medical Insurance Administration	37
17	Contribution	149	37	Supervision	54	57	Nanning City	37
18	Designated	144	38	Application	54	58	Contribution	37
19	Payment	125	39	Compliance	51	59	Proportion	36
20	Standard	117	40	Scope	49	60	Organization	36

It should be clearly noted that the high-frequency word analysis served only as an auxiliary reference for setting the PMC secondary variables. While it reflects the discursive emphasis of the policy texts, the final determination of variables was not derived from this analysis alone. The primary-level variables in this study were established following the research frameworks of Estrada (15), Zhang et al. (17), and Chen (18). The secondary variables were then defined based on the conceptual scope of the primary variables and adapted to the specific content of the LTCI policies. The high-frequency word analysis was used solely to validate whether the secondary variables adequately covered the core content dimensions of the LTCI policies, rather than serving as the sole or principal basis for variable selection.

Based on the above theoretical framework, this study established 9 primary-level variables and 40 secondary-level variables (see Table 3). The primary-level variables are: Policy Nature (X1), Policy Timeliness (X2), Policy Recipients (X3), Policy Functions (X4), Policy Content (X5), Policy Domains (X6), Policy Instruments (X7), Policy Oversight (X8), and Incentives and Constraints (X9). Among these, X1, X2, X3, and X7 assess policy quality from the perspective of policy characteristics, while X4, X5, X6, X8, and X9 evaluate the effectiveness of LTCI policies in terms of target coverage, policy standardization, instrument diversity, incentive multiplicity, and oversight comprehensiveness. Each secondary variable was assigned a binary value (1 if the criterion was met, 0 otherwise). Following the standard operational procedures of the PMC index model (15, 16), this study adopted an equal weighting approach. To assess the potential impact of weighting schemes on the evaluation results, this study additionally employed an alternative differentiated weighting scheme for sensitivity analysis, with the specific methods and results presented in Section 3.2.1. However, the equal weighting assumption—that each secondary variable contributes equally to overall policy quality—may not accurately reflect the differential impacts of various policy elements, which constitutes a limitation of this study.

1. Construction of the multi-input–output table

**Table 3:** Variable settings for long-term care insurance policy evaluation.

First-level variable	Second-level variable	Assignment criteria (1 = Yes, 0 = No)	Source or basis
X1 Policy Nature	X_1-1_ Description	The policy contains a substantive, context specific description of the LTCI system (local aging situation, policy rationale, or explicit positioning); not merely a single generic sentence.	Zhang Yong’an and Xi Haituo, Zhang et al. (17), Dong et al. (27)
	X_1-2_ Suggestion	The policy uses non binding language (“encourage,” “guide,” “explore”) without accompanying mandatory terms in the same clause; if a clause mixes “encourage” with “shall/must,” it is classified under X1-3.	
	X_1-3_ Guidance	The policy contains normative binding language (“shall,” “should,” “must,” “are required to”) that imposes obligations on specific actors.	
	X_1-4_ Support	The policy includes explicit resource commitment or support statements (e.g., fiscal subsidies, insurance fund support, tax concessions, land use priority) beyond general encouragement.	
	X_1-5_ Supervision	The policy stipulates supervision, inspection, performance evaluation, or penalty arrangements with identifiable responsible entities.	
X2 Policy Timeliness	X_2-1_ Short-term (≤3 years)	The policy explicitly states that a specific task, pilot phase, or quantitative target must be completed within ≤3 years, and provides a verifiable deadline or timeline.	Xue et al. (7), Zhang (16), Zhang et al. (17), Chen (18)
	X_2-2_ Mid-term (3–5 years)	The policy states mid term goals or institutional development milestones spanning 3–5 years, distinguishable from short term operational tasks.	
	X_2-3_ Long-term (≥5 years)	The policy contains provisions looking beyond 5 years (e.g., “by 2035…,” “long term planning,” “institutional finalization”), indicating a strategic horizon.	
X3 Policy Recipient	X_3-1_ Administrative Department	The policy assigns responsibilities, duties, or coordination roles to named administrative departments (medical insurance, finance, health, civil affairs, etc.).	Xiao et al. (8), Peng et al. (9), Qin et al. (19)
	X_3-2_ Care Institution	The policy specifies obligations, standards, access conditions, or service requirements for care institutions (nursing homes, hospitals, community care centers).	
	X_3-3_ Commercial insurance institution	The policy explicitly mentions commercial insurance institutions in roles such as underwriting, agency operation, fund management, or product development.	
	X_3-4_ Insured population	The policy defines the insured population (urban employees, residents, flexible workers, etc.) with enrollment rules, contribution obligations, or benefit eligibility criteria.	
X4 Policy Function	X_4-1_ Clarify standards	The policy establishes quantifiable or technical standards (e.g., disability assessment scales, payment caps, service frequency, facility to patient ratios), not merely behavioral guidelines.	Bu Lingtong, Zhang Jiawei (32)
	X_4-2_ Standard Guidance	The policy provides behavioral norms for actors (e.g., “insured persons shall truthfully declare…,” “institutions shall establish service archives”), distinct from technical standards.	
	X_4-3_ Clarify rights and responsibilities	The policy delineates the specific rights and responsibilities of at least two named stakeholder types (e.g., “the medical insurance bureau is responsible for…; the commercial insurer shall…”). Generic “all parties shall fulfill their duties” is insufficient.	
	X_4-4_ Categorized Regulation	The policy adopts different regulatory measures for different objects (e.g., separate rules for institutions vs. individuals vs. assessors), with identifiable category specific provisions.	
X5 Policy Content	X_5-1_ Beneficiaries	The policy defines the scope and written eligibility criteria for LTCI beneficiaries (e.g., “severely disabled for ≥6 months,” specific assessment score thresholds).	Sun et al. (6), Xue and Zhang (7), Zhang et al. (17)
	X_5-2_ Pooling model	The policy specifies the fund pooling level (municipal, provincial) and the corresponding revenue expenditure management framework.	
	X_5-3_ Division of functions	The policy describes a static allocation of functions across named departments (e.g., “Bureau A is responsible for X, Bureau B for Y”), without specifying interactive mechanisms (see X5-8 for those).	
	X_5-4_ Fund Management	The policy contains dedicated provisions on fund management: dedicated accounts, independent accounting, budget management, or fund operation analysis.	
	X_5-5_ Application Process	The policy delineates the step by step procedure for benefit application, disability assessment, review, and approval, not merely stating “application shall follow regulations.”	
	X_5-6_ Regulatory Operation	The policy specifies operational requirements for agencies via enforceable instruments (service agreements, performance evaluation criteria, renewal/termination conditions), not generic “strengthen management.”	
	X_5-7_ Accountability and Handling	The policy identifies concrete consequences for violations (e.g., warnings, fines, disqualification, fund recovery) linked to specific prohibited behaviors.	
	X_5-8_ Collaborative management	The policy mandates interactive coordination mechanisms across at least two departments (joint inspections, information sharing platforms, regular joint meetings). Keywords: “together with,” “jointly,” “data sharing,” “liaison meetings.”	
X6 Policy Field	X_6-1_ Economy	The policy addresses economic/financial dimensions: financing sources, payment standards, fiscal subsidies, cost sharing ratios, or fund balance mechanisms.	Zhang Yong’an and Xi Haituo and Dong et al. (27)
	X_6-2_ Social Services	The policy addresses service delivery dimensions: care services, older adults care institutions, community/home based care, or service quality monitoring.	
	X_6-3_ Technology	The policy mandates or describes information systems, intelligent platforms, tele care technology, or data sharing infrastructure as operational necessities.	
	X_6-4_ Politics	The policy references political/institutional hierarchy dimensions: policy level positioning, inter departmental command structures, or linkage with national reform agendas.	
	X_6-5_ System	The policy contains provisions that explicitly anchor itself within a higher legal/regulatory framework (e.g., “formulated in accordance with [Law X],” “these rules are supplementary to [National Guideline Y]”). Generic mention of “system” is insufficient.	
X7 Policy Instruments	X_7-1_ Supply-oriented	The policy directly expands service supply through specific measures: personnel training programs, institution construction plans, bed increase targets, or dedicated supply side funding lines.	Li and Jiang (10) and Rothwell (28)
	X_7-2_ Demand-oriented	The policy directly stimulates or channels demand through consumer subsidies, payment design, service vouchers, public procurement of services, or demand side eligibility expansions.	
	X_7-3_ Environmental	The policy indirectly influences supply/demand by establishing regulatory frameworks, information sharing environments, strategic plans, or risk management systems.	
X8 Policy Supervision	X_8-1_ Pre-supervision	The policy establishes ex ante control mechanisms: access review, qualification audit, agreement signing, pre service training requirements.	Chen and Zhang (29)
	X_8-2_ Concurrent supervision	The policy establishes ongoing monitoring mechanisms: routine inspections, dynamic monitoring, real time data reporting, cost auditing during service delivery.	
	X_8-3_ Post-supervision	The policy establishes ex post accountability mechanisms: violation handling, exit/termination rules, performance based penalties, public disclosure of results.	
X9 Incentive and Constraint	X_9-1_ Financial incentives and subsidies	The policy provides specific, quantified or procedurally defined fiscal subsidies, reward based grants, or operational subsidies to institutions/personnel/insurers. Mere “provide appropriate subsidies” without procedure or criteria is insufficient.	Zhang et al. (17), Fu et al. (30), Gao and Sun (31)
	X_9-2_ Legal protection	The policy cites superior laws/regulations that provide legal basis for the LTCI system, or includes legal safeguard clauses (e.g., “rights and obligations under this policy are protected by [Law]”).	
	X_9-3_ Personnel training	The policy mandates structured personnel development measures (vocational qualification programs, continuing education credits, government subsidized training slots), beyond generic “strengthen training.”	
	X_9-4_ Risk management	The policy institutes specific risk control mechanisms: fund balance early warning, internal auditing procedures, fraud reporting hotlines, information security protocols.	
	X_9-5_ Institutional coordination	The policy operationalizes coordination with adjacent systems (medical insurance, older adults care, social assistance, disability benefits) through concrete referral pathways, data sharing agreements, or joint service delivery protocols.	

The multi-input–output table (Table 4) presents the correspondence between the 9 primary-level variables and their respective secondary variables. Each secondary variable was assigned a binary value: 1 if the policy text met the evaluation criterion for that variable, and 0 otherwise. The score for each primary-level variable was calculated as the arithmetic mean of the scores of its constituent secondary variables.

1. Calculation of the PMC Index

**Table 4:** The multi-input–output table.

First-level variable	Second-level variable
X1 Policy Nature	X1-1、X1-2、X1-3、X1-4、X1-5
X2 Policy Timeliness	X2-1、X2-2、X2-3
X3 Policy Recipient	X3-1、X3-2、X3-3、X3-4
X4 Policy Function	X4-1、X4-2、X4-3、X4-4
X5 Policy Content	X5-1、X5-2、X5-3、X5-4、X5-5、X5-6、X5-7、X5-8
X6 Policy Field	X6-1、X6-2、X6-3、X6-4、X6-5
X7 Policy Instruments	X7-1、X7-2、X7-3
X8 Policy Supervision	X8-1、X8-2、X8-3
X9 Incentive and Constraint	X9-1、X9-2、X9-3、X9-4、X9-5

The second-level variables in the multi-input–output table were coded according to Equations 3 and 4. Next, the values of the first-level variables were calculated using Equation 5. Finally, the PMC index for each policy was derived using Equation 6, where *t* and *j* denote first-level and second-level variables, respectively; *t* denotes the number of first-level variables and *n* denotes the number of second-level variables.


X∼N[0,1] (3)

X={XR:[0,1]} (4)

$$X_{t}=(\sum_{j=1}^{n}\frac{X_{t-j}}{T(X_{t-j})})\;\;\;\;\;\;\;t=1,2,3...$$ (5)

$$\mathrm{PMC}=[\begin{array}{l} X_{1}(\sum_{j=1}^{5}\frac{X_{1-j}}{5})+X_{2}(\sum_{j=1}^{3}\frac{X_{2-j}}{3})+X_{3}(\sum_{j=1}^{4}\frac{X_{3-j}}{4})+\\ X_{4}(\sum_{j=1}^{4}\frac{X_{4-j}}{4})+X_{5}(\sum_{j=1}^{8}\frac{X_{5-j}}{8})+X_{6}(\sum_{j=1}^{5}\frac{X_{6-j}}{4})+\\ X_{7}(\sum_{j=1}^{3}\frac{X_{7-j}}{3})+X_{8}(\sum_{j=1}^{3}\frac{X_{8-j}}{3})+X_{9}(\sum_{j=1}^{5}\frac{X_{9-j}}{5}) \end{array}]$$ (6)

The PMC index is divided into four grades: a score of 9 indicates a perfect policy; 7–8.99 indicates excellent; 5–6.99 indicates good; and a score ≤4.99 indicates poor (see Table 5).

1. Plotting the PMC surface chart

**Table 5:** LTCI policy rating scale.

PMC Index score	0 ~ 4.99	5 ~ 6.99	7 ~ 8.99	9
Grade	Poor	Good	Excellent	Perfect

Based on the PMC indices calculated above, the PMC surfaces of long-term care insurance policies at different grades can be plotted using Equation 7. Herein, the specific values of the PMC indices are represented by the vertical axis, and the different dimensions of the first-level variables X1-X9 are represented by the planar coordinates.


$$P=(\begin{array}{ccc} \mathrm{X1} & \mathrm{X2} & \mathrm{X3}\\ \mathrm{X4} & \mathrm{X5} & \mathrm{X6}\\ \mathrm{X7} & \mathrm{X8} & \mathrm{X9} \end{array})$$ (7)

### 2.3 Statistical methods

Data collation and descriptive analysis were performed using WPS Office. Text mining was conducted using ROSTCM6. The heatmaps for the two-dimensional analysis of policy instruments and stakeholders were generated using RStudio (version 2022.02.3 + 492, R 4.4.3).

## 3 Results

### 3.1 Policy text content analysis

For the nine core policy texts from the five cities, two coders independently performed coding following the “policy number–specific chapter–clause sequence” format. A sample of the coding results is presented in Table 6. After the reliability test, the inter-coder agreement rate (Holsti coefficient *R*) was 0.9866. Discrepant items were discussed and resolved by consensus, yielding a total of 598 valid policy instrument coding units.

**Table 6:** Policy text content analysis coding scheme (excerpt).

Policy name	Content analysis unit	Coding number	Policy instrument	Stakeholder
Notice on Printing and Distributing the Pilot Work Plan for the Long-term Care Insurance System of Hezhou City	Strive to basically establish a long-term care insurance system that adapts to the economic and social development of the city and meets the needs of the people during the “13th Five-Year Plan” period.	P1(1)-2–1	Environment-oriented—strategic planning	Administrative departments
	During the pilot phase, the long-term care insurance fund shall be financed through the transfer of funds from the urban employee basic medical insurance pooling fund, the major illness assistance medical insurance fund, and individual contributions.	P1(1)-3–4	Supply-oriented—funding supply	Insured individuals
	...	...	...	...
Notice on Printing and Distributing the Implementing Rules for the Long-term Care Insurance System of Hezhou City (Trial)	Establish risk management mechanisms such as whistleblowing and complaints, information disclosure, internal controls, and fraud prevention to ensure the balanced operation, security, and reliability of the fund.	P1(2)-6–3	Environment-oriented—risk management	Administrative departments
	...	...	...	...
Implementing Opinions on Promoting the Long-term Care Insurance System of Nanning City	Encourage and support commercial insurance institutions to develop and customize long-term care insurance products for urban and rural residents, and promote the establishment and improvement of a multi-tiered long-term care security system that meets the diverse needs of the public.	P2(1)-2–4	Environment-oriented—strategic planning	Commercial insurers
	The long-term care insurance fund shall be raised primarily through contributions from employers and insured individuals, fiscal subsidies, and shall accept social donations.	P2(1)-3–5	Supply-oriented—funding supply	Insured enterprises, insured individuals, social donors
	...	...	...	...
...	...	...	...	...
Notice on Printing and Distributing the Implementation Plan for Establishing a Long-term Care Insurance System in Guigang City	The tax authorities shall be responsible for collecting long-term care insurance premiums and shall cooperate with the healthcare security authorities and financial authorities in fundraising efforts.	P5-6-8	Environment-oriented—regulatory control	Administrative departments
	The market regulatory authorities shall be responsible for price supervision of nursing service items and assistive device rentals, and shall investigate and penalize improper pricing practices such as failure to clearly mark prices, price fraud, and market price manipulation.	P5-6-9	Environment-oriented—collaborative oversight	Administrative departments

#### 3.1.1 Overall distribution of policy instruments

In terms of the distribution across the three major instrument types (see Table 7), environment-oriented instruments accounted for the largest proportion (41.47%, 248 items), followed by demand-oriented instruments (33.44%, 200 items), while supply-oriented instruments represented the smallest share (25.08%, 150 items). Among the 12 specific instrument subcategories, payment provisions (127 items, 21.24%), regulatory control (115 items, 19.23%), and collaborative oversight (78 items, 13.04%) ranked as the top three, together accounting for 53.51% of all instruments. Facility construction (6 items, 1.00%), social donations (0 items), and workforce development (16 items, 2.68%) were severely underrepresented.

**Table 7:** Distribution of policy instruments by type.

Instrument type	Node count (%)	Sub-instrument	P1	P2	P3	P4	P5	Total	Proportion
Supply-oriented	150 (25.08%)	Funding supply	20	17	16	15	8	76	12.71%
		Infrastructure development	6	0	0	0	0	6	1.00%
		Human resource development	10	2	1	2	1	16	2.68%
		Standard setting	26	7	7	9	3	52	8.70%
Demand-oriented	200 (33.44%)	Public procurement	9	7	3	4	1	24	4.01%
		Service outsourcing	18	5	3	2	1	29	4.85%
		Consumption subsidies	7	2	5	2	4	20	3.34%
		Payment design	38	23	13	30	23	127	21.24%
Environment-oriented	248 (41.47%)	Regulatory control	36	14	12	42	11	115	19.23%
		Strategic planning	11	7	6	9	5	38	6.35%
		Collaborative supervision	26	16	11	20	5	78	13.04%
		Risk management	4	2	3	4	4	17	2.84%
Total	598 (100%)	——	211	102	80	139	66	598	100.00%

In terms of city-level distribution, P1 (Hezhou) had the highest total number of policy instruments (211 items, 35.28%), while P5 (Guigang) had the lowest (66 items, 11.04%). P1 also ranked first among the five cities in both supply-oriented instruments (62 items) and environment-oriented instruments (77 items). P4 (Liuzhou) had the highest proportion of demand-oriented instruments (38 items, accounting for 27.34% of its total instruments).

#### 3.1.2 Policy instrument–stakeholder interaction analysis

The distribution of policy instruments across stakeholder categories is presented in Table 8. From the perspective of supply-oriented instruments, administrative departments were the dominant actors (43.33%, 65/150), followed by insured individuals (25.33%, 38/150), and care service institutions (13.33%, 20/150), with the latter primarily concentrated in workforce development (6 items) and standard-setting (12 items). Beneficiaries accounted for only 1.33% (2/150) of supply-oriented instruments, both of which fell under the “standard-setting” subcategory. This reflects that the current design of supply-oriented instruments has not yet established a demand-feedback mechanism from the beneficiary side to the supply side—that is, beneficiaries have not been incorporated into the policy framework for supply-side decisions regarding funding allocation, facility construction, or workforce development.

**Table 8:** Two-dimensional cross-analysis of LTCI policy instruments and stakeholders.

Type	Sub-instrument	Insured unit	Insured individual	Beneficiary group	Care service institution	Commercial insurance institution	Social donor	Disability assessment institution	Administrative department	Total
Supply-oriented	Funding supply	12	35	0	0	1	5	2	21	76
	Infrastructure development	0	0	0	2	1	0	0	3	6
	Human resource development	0	0	0	6	0	0	0	10	16
	Standard setting	0	3	2	12	0	0	4	31	52
Demand-oriented	Public procurement	0	0	1	4	9	0	4	6	24
	Service outsourcing	0	0	0	0	20	0	0	9	29
	Consumption subsidies	0	0	20	0	0	0	0	0	20
	Payment design	7	40	70	6	1	0	0	3	127
Environment-oriented	Regulatory control	9	13	10	29	1	0	8	45	115
	Strategic planning	0	2	3	4	3	1	0	25	38
	Collaborative supervision	0	0	2	1	3	0	1	71	78
	Risk management	0	0	0	0	0	0	0	17	17
	Total	28	93	108	64	39	6	19	241	598
	Proportion	4.68%	15.55%	18.06%	10.70%	6.52%	1.00%	3.18%	40.30%	100.00%

With respect to demand-oriented instruments, beneficiaries constituted the highest proportion (45.50%, 91/200), concentrated in payment provisions (70 items) and consumption subsidies (20 items), indicating that demand-side policies primarily protect beneficiaries through eligibility determination and cost reimbursement. Insured individuals ranked second (20.00%, 40/200), also concentrated in payment provisions. Commercial insurance institutions accounted for 15.00% (30/200), mainly in service outsourcing (20 items). Social donors were entirely absent from demand-oriented instruments (0/200).

In the environmental instrument category, administrative departments dominated with 63.71% (158/248), concentrated in regulatory control (45 items) and collaborative oversight (71 items). Care service institutions ranked second (13.71%, 34/248), with regulatory control (29 items) as their primary mode of interaction. Social donors accounted for the lowest share (0.40%, 1/248).

At the stakeholder level, administrative departments were the most central stakeholder group (40.30%, 241/598), followed by beneficiaries (18.06%, 108/598) and insured individuals (15.55%, 93/598). Social donors represented only 1.00% (6/598) of all stakeholder interactions, the lowest among all stakeholder categories.

#### 3.1.3 Heatmap visualization analysis

**Figure 2:**
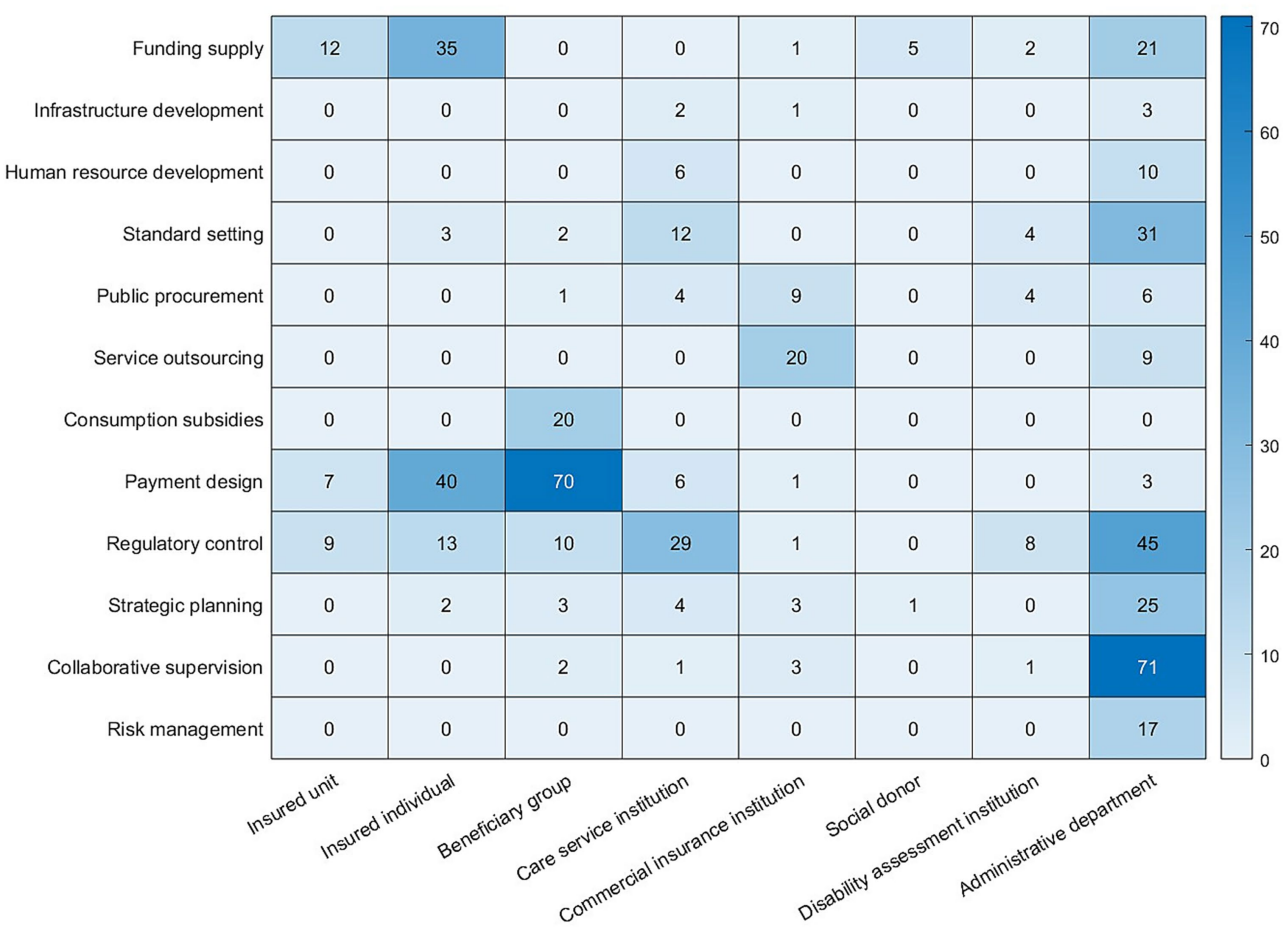
Heatmap of LTCI Policy Instruments – Stakeholder. Rows represent stakeholder categories, columns represent policy instrument subcategories, and color intensity reflects the frequency of interactions. The color gradient from light to dark indicates increasing interaction frequency, with dark blue areas denoting high-frequency interactions and white areas denoting low-frequency or no interactions.

The heatmap in Figure 2 visually corroborates the quantitative findings presented above.

First, the darkest areas are concentrated at the intersections of “administrative departments–collaborative oversight” (71) and “administrative departments–regulatory control” (45), indicating that administrative oversight and rule-making constitute the core of the current policy system, with the policy logic predominantly characterized by “government-driven” and “rule-based” approaches.

Second, the color distribution in the row for “beneficiaries” is highly skewed: of the 108 interactions, 70 are concentrated in “payment provisions” (accounting for 64.81%), while supply-oriented instruments (funding supply 0, workforce development 0, facility construction 0) and “risk management” within environment-oriented instruments (0) are entirely blank, with only 2 interactions recorded for “collaborative oversight.” This suggests that policy design essentially equates beneficiary protection with financial compensation, while neglecting capacity building, quality supervision, and channels for demand expression.

Third, the row for “social donors” is almost entirely blank (with only 6 interactions), and “commercial insurance institutions” are primarily associated with “service outsourcing” (19) and “public procurement” (9), rather than participating in strategic roles such as policy design or quality assurance. The marginalization of non-governmental actors is clearly evident.

### 3.2 PMC index model evaluation results

To ensure the scientific rigor and reproducibility of the policy evaluation process, two coders independently assigned scores to 40 secondary variables across the five cities (200 items in total) following the PMC coding manual. After the scoring was completed, Cohen’s Kappa coefficient was calculated using SPSS to assess inter-coder reliability, yielding a value of 0.918 (*p* < 0.001), indicating excellent agreement. Items with discrepancies were discussed and resolved by consensus based on the evaluation criteria specified in the coding manual, resulting in the final PMC scoring dataset. The scoring results are presented in Table 9.

**Table 9:** Scoring results.

First-level variable	Second-level variable	P1	P2	P3	P4	P5
X1 Policy Nature	X1-1 Description	1	0	1	0	0
	X1-2 Suggestion	1	1	1	1	1
	X1-3 Guidance	1	1	1	1	1
	X1-4 Support	0	0	1	0	0
	X1-5 Supervision	1	1	1	1	1
X2 Policy Validity	X2-1 Short-term	0	0	1	1	1
	X2-2 Mid-term	1	1	0	1	0
	X2-3 Long-term	0	0	0	0	0
X3 Policy Recipient	X3-1 Administrative Department	1	1	1	1	1
	X3-2 Care Institution	1	1	1	1	1
	X3-3 Commercial Insurance	1	1	1	1	1
	X3-4 Insured Population	1	1	1	1	1
X4 Policy Function	X4-1 Clarify Standards	1	1	1	1	1
	X4-2 Standard Guidance	1	1	1	1	1
	X4-3 Clarify Rights	1	1	1	1	1
	X4-4 Categorized Regulation	0	0	0	0	0
X5 Policy Content	X5-1 Beneficiaries	1	1	1	1	1
	X5-2 Pooling Model	1	1	1	1	1
	X5-3 Division of Functions	1	1	1	1	1
	X5-4 Fund Management	1	1	1	1	1
	X5-5 Application Process	1	1	1	1	0
	X5-6 Regulatory Operation	1	1	1	1	1
	X5-7 Accountability	0	1	1	0	0
	X5-8 Collaborative Management	1	1	1	1	1
X6 Policy Fields	X6-1 Economy	1	1	1	1	1
	X6-2 Social Services	1	1	0	1	0
	X6-3 Technology	1	1	1	1	1
	X6-4 Politics	1	1	1	1	1
	X6-5 System	1	1	1	1	0
X7 Policy Tools	X7-1 Supply-oriented	1	0	0	0	0
	X7-2 Demand-oriented	1	1	1	1	1
	X7-3 Environmental	1	1	1	1	1
X8 Policy Supervision	X8-1 Pre-supervision	1	1	0	0	0
	X8-2 Concurrent Supervision	1	1	1	1	1
	X8-3 Post-supervision	0	1	1	0	0
X9 Incentive Constraints	X9-1 Financial Incentives	1	0	0	0	0
	X9-2 Legal Protection	1	1	1	1	0
	X9-3 Personnel Training	1	1	1	1	0
	X9-4 Risk Management	1	1	1	1	1
	X9-5 Institutional Coordination	0	0	0	0	0

#### 3.2.1 PMC index scores

Based on the scoring table and the calculation formulas, the PMC index scores for the LTCI policies of the five cities—Hezhou, Nanning, Liuzhou, Beihai, and Guigang—were derived (see Table 10). The mean PMC index score across the five cities was 6.59 (rated as “good”). Among them, P1 (Hezhou) recorded the highest score (7.23, rated as “excellent”), followed by P3 (Beihai) with 7.07 (also “excellent”), while P2 (Nanning, 6.95), P4 (Liuzhou, 6.49), and P5 (Guigang, 5.23) were all rated as “good.” In terms of the mean scores of the primary-level variables, Policy Recipients (X3, mean = 1.00), Policy Domains (X6, mean = 0.88), and Policy Content (X5, mean = 0.90) scored relatively high, indicating that the five cities’ policies performed well in terms of beneficiary coverage, domain relevance, and content completeness. Policy Timeliness (X2, mean = 0.40) and Incentives and Constraints (X9, mean = 0.56) scored the lowest, representing the core weaknesses constraining policy quality improvement.

**Table 10:** Scores of long-term care insurance policies.

Policy code	X1	X2	X3	X4	X5	X6	X7	X8	X9	PMC index	Grade
P1	0.80	0.33	1.00	0.75	0.88	1.00	1.00	0.67	0.80	7.23	Excellent
P2	0.60	0.33	1.00	0.75	1.00	1.00	0.67	1.00	0.60	6.95	Good
P3	1.00	0.33	1.00	1.00	1.00	0.80	0.67	0.67	0.60	7.07	Excellent
P4	0.60	0.67	1.00	0.75	0.88	1.00	0.67	0.33	0.60	6.49	Good
P5	0.60	0.33	1.00	0.75	0.75	0.60	0.67	0.33	0.20	5.23	Good
Mean	0.72	0.40	1.00	0.80	0.90	0.88	0.73	0.60	0.56	6.59	Good

More specifically, P1 (Hezhou) achieved an “excellent” rating with a score of 7.23. As the second batch of pilot cities nationwide and the first pilot city in Guangxi, Hezhou has accumulated considerable experience. The *Pilot Work Plan for the Long-term Care Insurance System of Hezhou City*, issued in 2018, clearly defined core elements such as the scope of coverage, financing mechanisms (with 70% borne by the employee medical insurance pooling fund and 10% by individuals), and payment standards (40 CNY per bed-day for home-based care, 50 CNY for institutional care, and 60 CNY for hospital care). The implementing rules further standardized the entire process including disability assessment, benefit payment, and service settlement. Hezhou’s high scores in Policy Instruments (X7) and Policy Nature (X1) are directly attributable to the institutional completeness resulting from its first-mover advantage in the pilot program. However, its score in Policy Timeliness (X2) was relatively low, as the policy only involved mid-term planning (X2-2) and lacked short-term and long-term planning, consistent with the statement in its policy document that “financing methods shall be separately adjusted and formulated after the conclusion of the pilot phase.”

P3 (Beihai) ranked second with an “excellent” score of 7.07. Beihai achieved full scores in Policy Nature (X1), Policy Functions (X4), and Policy Content (X5), though there remains room for improvement in Policy Timeliness (X2) and Incentives and Constraints (X9).

P5 (Guigang) recorded the lowest score among the five cities at 5.23. As the latest city to launch the pilot program (December 2025), Guigang scored the lowest among the five cities in Policy Domains (X6), Policy Supervision (X8), and Incentives and Constraints (X9), with notable gaps in its policy texts regarding financial incentives, legal protection, and workforce development. It is worth noting that Guigang was the only city among the five to have issued just one core policy document (the other cities each issued two). The limited number of policy documents objectively restricted the coverage of its policy content, which partially explains its relatively low PMC score.

To assess the robustness of the policy rankings, we conducted a sensitivity analysis using two different weighting schemes. In the first scheme, all 40 secondary indicators were assigned equal weights (1/40 = 0.025). In the second scheme, differentiated weights were assigned according to indicator importance: the first 10 indicators received a weight of 0.03, while the remaining 30 indicators received a weight of 0.0233 (summing to 1). The results show that the relative rankings of the five cities’ policies are highly consistent across both schemes (Table 11), with P1, P4 and P5 holding perfectly stable positions. Only P2 and P3 swapped between the second and third ranks, a variation well within an acceptable range. This indicates that the relative ordering of policy quality across the five cities is not substantially altered by changes in weight assignment, confirming the robustness of the evaluation results.

**Table 11:** Sensitivity analysis of policy rankings under different weighting schemes.

Policy	Equal weights	Alternative weights	Rank (equal weights)	Rank (alternative weights)
P1	0.83	0.82	1	1
P2	0.80	0.79	2	3
P3	0.80	0.80	3	2
P4	0.75	0.75	4	4
P5	0.60	0.60	5	5

#### 3.2.2 PMC surface diagram evaluation results

**Figure 3:**
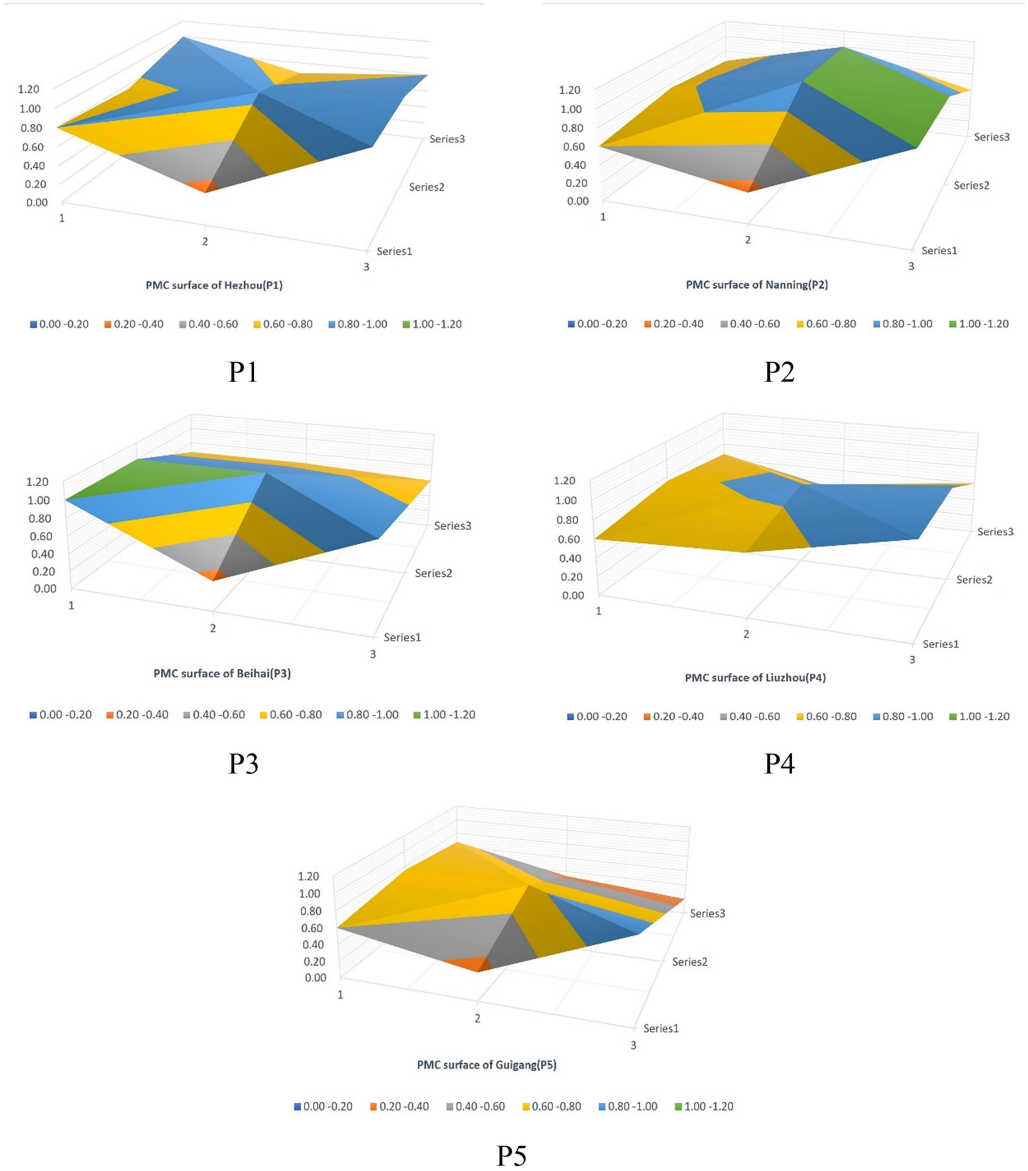
PMC Surface Diagrams of LTCI Policies (P1-P5).

**Figure 4:**
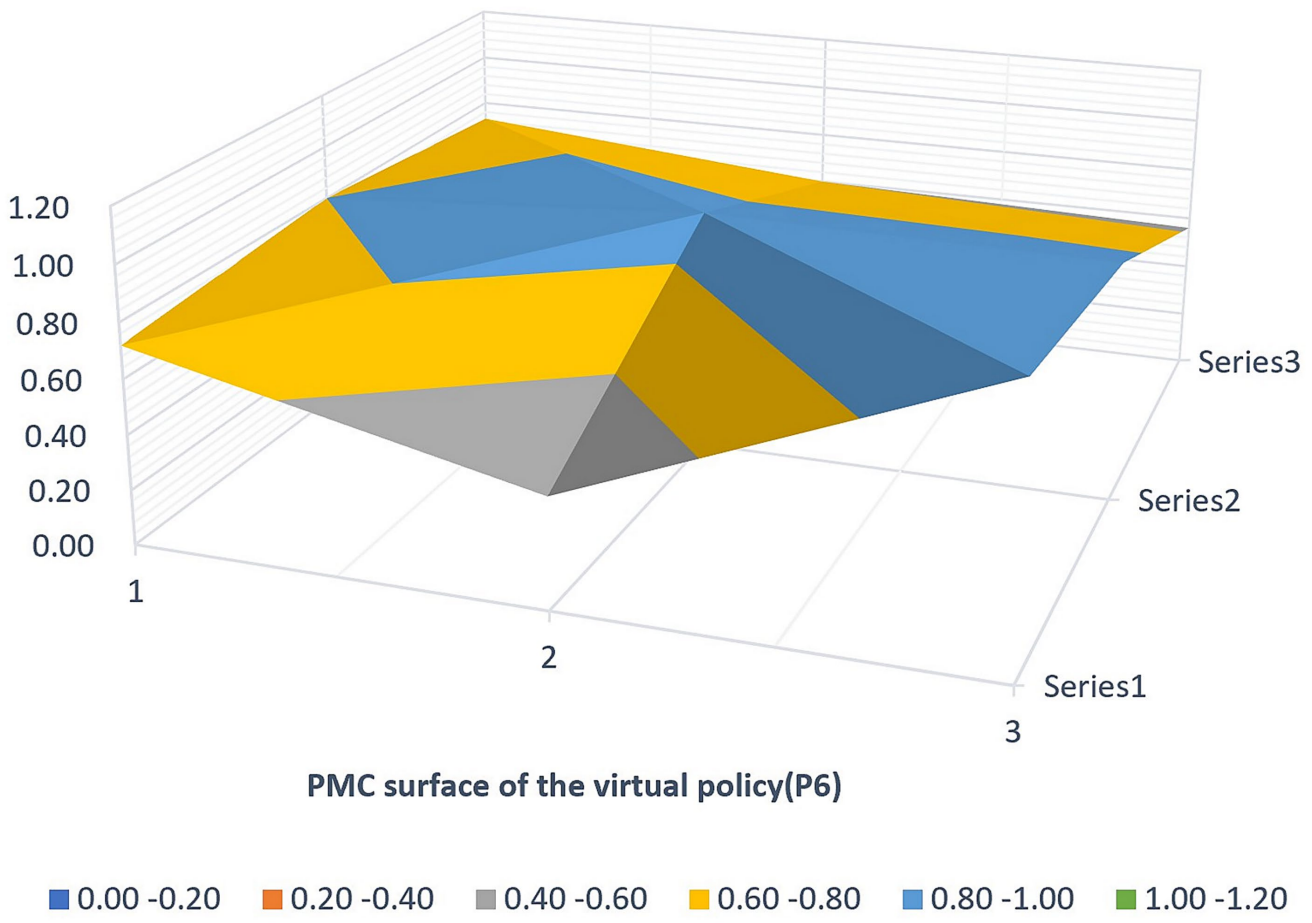
PMC Surface Diagram of the Virtual Policy P6.

To more intuitively present the distribution characteristics of each city’s policy scores across the nine primary-level variable dimensions, PMC surface diagrams were plotted based on the data in Table 9 (Figure 3). In addition, a hypothetical policy P6 was constructed using the mean values of all primary-level indicators to generate a reference PMC surface diagram (Figure 4).

As shown in Figure 3, the surface of P1 (Hezhou) exhibited a pronounced overall convexity, with only X2 (0.33) showing a concave dip. Compared to P6, X6 (1.00 vs. 0.88, +0.12) and X7 (1.00 vs. 0.73, +0.27) were significantly above the mean. This surface morphology corroborates Hezhou’s first-mover advantage as the inaugural pilot city in Guangxi in terms of institutional framework development. However, the dip in X2 reflects the limitation of early-stage pilot programs that prioritized immediate implementation over long-term planning.

The surface of P3 (Beihai) was relatively balanced, with concavities concentrated at X2 (0.33) and X9 (0.60). Compared to P6, X1 (1.00 vs. 0.72, +0.28) and X4 (1.00 vs. 0.80, +0.20) were substantially above the mean. This indicates that while Beihai’s policies achieved relatively complete horizontal coverage, gaps remain in the vertical dimensions of timeliness and incentive mechanisms.

The surface of P2 (Nanning) displayed multi-dimensional concavities. Compared to P6, X5 (1.00 vs. 0.90, +0.10) and X8 (1.00 vs. 0.60, +0.40) were significantly above the mean, while X7 (0.67 vs. 0.73, −0.06) was slightly below. The concavity in X7 stemmed from the absence of supply-oriented policy instruments (X7-1), as the policy did not explicitly address supply-side elements such as financial investment, organizational development, and personnel training. Nanning’s surface profile is characterized by “broad coverage with insufficient instrument balance.”

The surface of P4 (Liuzhou) displayed its most pronounced concavity at X8 (0.33). Compared to P6, X2 (0.67 vs. 0.40, +0.27) was significantly above the mean, while X8 (0.33 vs. 0.60, −0.27) was significantly below. Although Liuzhou led among the five cities in temporal planning, the absence of pre-supervision (X8-1 = 0) and post-supervision (X8-3 = 0)—i.e., a lack of effective control at the entry and exit points of policy implementation—made it the city with the largest gap in the supervision dimension relative to the mean.

The surface of P5 (Guigang) exhibited the widest range of concavities. Compared to P6, five dimensions were below the mean: X1 (0.60 vs. 0.72, −0.12), X5 (0.75 vs. 0.90, −0.15), X6 (0.60 vs. 0.88, −0.28), X8 (0.33 vs. 0.60, −0.27), and X9 (0.20 vs. 0.56, −0.36). Guigang showed systematic gaps in the dimensions of incentive mechanisms, policy domains, and policy supervision relative to the average of the five cities, reflecting that it remains at a preliminary “framework-building” stage.

## 4 Discussion

### 4.1 Main findings and interpretation

#### 4.1.1 Overall policy quality: initial framework established but long-term design insufficient

The mean PMC index score for the five Guangxi cities was 6.59 (rated as “good”), with Hezhou and Beihai achieving “excellent” ratings. The notably high scores in Policy Recipients and Policy Domains indicate that the basic institutional framework has been established. This assessment is further supported by implementation data. According to the official portal of the Guangxi Zhuang Autonomous Region People’s Government, by the end of 2025, the total number of LTCI enrollees in the region had reached 4.2344 million, with cumulative fund expenditures of 942 million CNY and a cumulative total of 26,100 individuals having received benefits (20). As the first pilot city, Nanning had covered 2.2714 million people by July 2025, with more than 23,300 disabled individuals having accessed various forms of social care services, including institutional care, home-based care, and assistive device rental services. The annual per capita financial burden reduction attributable to the system exceeded 20,000 CNY (21, 22). In 2024, Beihai became one of the first cities nationally to transition from “steady pilot advancement” to “comprehensive establishment” of the LTCI system, taking the lead in Guangxi to achieve full coverage for both employees and residents. These data indicate that Guangxi’s LTCI policies have not only completed the institutional framework at the textual level but have also begun to generate preliminary burden-reduction effects in practice.

However, the pronounced concavities in X2 (Policy Timeliness) and X9 (Incentives and Constraints) suggest that while the basic institutional framework is in place, “long-term planning” and “incentive mechanisms” have not yet been embedded at the core of policy design. None of the cities have established long-term plans (X2-3 = 0 for all), and policy incentives are generally absent (only P1 received a score). At the regional level, Guangxi is advancing full LTCI coverage in three phases: exceeding 5 million enrollees in 2025, projected to exceed 10 million in 2026, and achieving basic full coverage by the end of 2028 (20). Although this phased arrangement reflects a progressive expansion logic, the policy texts themselves lack systematic elaboration of long-term institutional objectives across the five cities. While Policy Recipients coverage (X3 = 1.00) and Policy Domains alignment (X6 = 0.88) meet the basic requirements of the national framework, the low scores in Policy Timeliness and Incentives and Constraints expose a critical gap in ethnic minority regions between institutional framework establishment and long-term operational sustainability—that is, while the basic institutional framework has been established, sustainable financing mechanisms and incentive–constraint mechanisms have yet to be fully integrated into policy design.

#### 4.1.2 Policy instrument structure: environment-oriented dominance and supply-side weakness

The five cities exhibited a pronounced “environment-oriented dominance” (41.47%), with supply-oriented (25.08%) and demand-oriented (33.44%) instruments accounting for relatively lower proportions. This finding is consistent with existing research; Li and Jiang (10) similarly identified structural imbalances in the use of policy instruments in their analysis of local LTCI pilot policies, finding that environment-oriented instruments were overused while supply- and demand-oriented instruments were relatively underrepresented.

The overuse of environment-oriented instruments—with regulatory control and collaborative oversight together accounting for 77.82% of all environment-oriented instruments—reflects the pathway choice of late-pilot cities: rapidly establishing the institutional framework through administrative means. While this strategy is justifiable during the initial phase of institutional development, over-reliance may lead to excessive regulatory density and inhibit market vitality (23).

The weakness of supply-oriented instruments—with only 6 items for facility construction and 16 for workforce development—represents a distinguishing feature of Guangxi compared to the national average. Facility construction was exclusively concentrated in P1 (Hezhou), with the remaining four cities scoring zero. For ethnic minority regions with weak care infrastructure, the absence of long-term resource input mechanisms for facilities and personnel will severely constrain service accessibility. Notably, although “workforce development” appeared only 16 times in the policy texts, according to reports from Guangxi News Network, Nanning had already trained 9,471 certified long-term care service personnel through the LTCI system and attracted 21 home-based care service chain institutions—with proven operational experience in first-tier cities—to establish their presence in Nanning for the first time (24). This suggests that the implementation effects of policy instruments may be amplified through market mechanisms, and that policy text analysis cannot fully capture such indirect effects. It also indicates that low frequency of a particular instrument in policy texts does not necessarily imply a substantive gap in that domain.

The structural simplification of demand-oriented instruments—with payment provisions accounting for 63.50% of all demand-oriented instruments, while consumption subsidies and public procurement remained limited—indicates that policies tend to manage demand through eligibility criteria rather than directly activating markets through fiscal subsidies or government purchasing of services. This is consistent with a general characteristic of LTCI pilots: most pilot cities prioritize addressing the question of “who is eligible for benefits” during the initial institutional phase, while paying insufficient attention to “how to expand service supply through market-oriented approaches” (11).

The dominance of environment-oriented instruments and the weakness of supply- and demand-oriented instruments fundamentally reflect Guangxi’s pathway choice as a late-developing ethnic minority region: under conditions of underdeveloped care service markets and limited fiscal capacity, the primary task of policy-making is to establish institutional rules rather than to expand service capacity. While this strategy is reasonable during the initial institutional phase, it also implies that Guangxi faces a longer development cycle than more developed regions in advancing the national policy objectives of “enhancing care service supply capacity” and “activating market demand.”

#### 4.1.3 Stakeholder participation: administrative dominance and pluralistic absence

Administrative departments were the absolute center of stakeholder participation (40.30%), accounting for 43.33% of supply-oriented instruments and 63.71% of environment-oriented instruments. Although beneficiaries constituted the highest proportion in demand-oriented instruments (45.50%), their participation was heavily concentrated in payment provisions, while they accounted for only 1.33% of supply-oriented instruments. This suggests that the policy treats disabled individuals merely as passive recipients, incorporating them into the framework of financial compensation while excluding them from policy design related to capacity building, quality oversight, and demand articulation.

Social donor participation was extremely low (1.00%) and entirely absent from demand-oriented instruments, standing in stark contrast to the policy rhetoric of “encouraging social donations.” The six strategic planning items lacked actionable supporting measures. This pattern of ideas without mechanisms indicates that in ethnic minority regions, social force mobilization remains at the aspirational stage and has yet to be institutionalized. Han and Chen (25) observed that various stakeholders in LTCI hold different interests and that a balance of interests must be achieved to form policy synergy. Our findings further reveal the extent of this imbalance: beneficiaries are nearly absent from decisions regarding resource allocation, and social forces are entirely excluded from demand-side institutional design.

The administratively driven governance structure is consistent with the national framework of “government-led, pluralistic participation.” However, the fact that social donors account for only 1.00% and beneficiaries are excluded from supply-side decisions reflects the practical constraints that Guangxi faces in cultivating diverse actors—namely, underdeveloped social forces and immature market mechanisms—which means that “pluralistic participation” currently remains at the level of strategic planning and has not yet been translated into actionable institutional arrangements.

#### 4.1.4 Characterization of the “Guangxi model”

Synthesizing the quantitative analysis and policy implementation data presented above, the core features of the “Guangxi model” as reflected in the region’s LTCI policies can be summarized across three dimensions.

First, a progressive coverage pathway. From Nanning’s early pilot in 2021, to Beihai’s achievement of full employee and resident coverage in 2024, to the establishment of the system across six coordination zones region-wide with 4.2344 million enrollees by the end of 2025 (20), Guangxi has followed a progressive trajectory from pilot to expansion and from employee-based to resident-based coverage. According to the official portal of the Guangxi Zhuang Autonomous Region People’s Government, the region is advancing in three phases, with projections exceeding 10 million enrollees in 2026 and achieving basic full coverage by the end of 2028 (26). This pathway aligns with the fiscal affordability of Guangxi as an economically less-developed ethnic minority region; the phased approach mitigates excessive pressure on fund expenditures in the short term while allowing time for the gradual cultivation of care service capacity.

Second, a multi-layered benefit coordination mechanism. Guangxi has promoted the combined use of older adults care service consumption vouchers and LTCI benefits, with a monthly subsidy of up to 800 CNY, allowing eligible beneficiaries to offset 50% of home-based care costs and 40% of institutional care costs following LTCI benefit receipt. Additionally, the family physician contract coverage rate for residents aged 65 and above has reached 73.6% (26). This multi-layered benefit design, which coordinates multiple policy instruments to achieve synergistic effects with relatively limited fiscal input, represents a pragmatic response to the uneven economic development levels and varying affordability across ethnic minority regions.

Third, an administratively driven, regulation-oriented governance structure. Environment-oriented instruments accounted for 41.47%, and administrative departments accounted for 40.30% of stakeholder interactions, depicting a landscape in which “the government constructs the institutional framework through intensive rule-making.” Under conditions of underdeveloped care service markets and limited social force participation capacity, the pragmatic choice has been to “establish institutional rules first, and then build service capacity.” The high scores in Policy Recipients coverage (X3 = 1.00) and Policy Domains alignment (X6 = 0.88) indicate that the five cities’ policies have met the national framework requirements of “ensuring basic needs with wide coverage” in terms of beneficiary scope and coverage. However, the persistently low scores in Policy Timeliness (X2 = 0.40) and Incentives and Constraints (X9 = 0.56), together with the gap between 4.2344 million enrollees and 26,100 benefit recipients (a benefit receipt rate of only 0.62%) (20), reveal a phased gap between this pathway and the national long-term goal of “establishing a stable and sustainable LTCI system.” The “Guangxi model” has completed the transition from having no institutional framework to having one in place, but it has not yet achieved the transition from “having institutions” to “achieving effective outcomes,” nor from “government-led” to “pluralistic co-governance.”

### 4.2 Policy recommendations

#### 4.2.1 Promoting the county-level distribution of nursing facilities and care workforce

Supply-oriented instruments in Guangxi accounted for only 25.08%, with facility construction appearing in only 6 items—all concentrated in Hezhou and entirely absent in the other four cities—and workforce development in only 16 items. It is recommended that nursing facilities be incorporated into each city’s healthcare security development plan, with small-scale nursing stations deployed at the county level, prioritizing ethnic townships and border towns. A tiered and categorized care workforce training mechanism should be established: standardized vocational skills training for institutional care providers, and basic care skills training for rural surplus labor and family members of disabled individuals, with vocational training subsidies incentivizing certified personnel to remain in county-level services. Nanning has already trained nearly 10,000 individuals and attracted 21 chain institutions to establish a presence through market mechanisms (21), indicating a spontaneous market response. However, this practice has not yet been translated into systemic institutional arrangements in policy texts. It is recommended that the healthcare security departments of each city draw on Nanning’s experience and develop complementary implementation rules for care workforce training and incentives.

#### 4.2.2 Optimizing the internal structure of demand-oriented policy instruments

Payment provisions accounted for 63.50% of demand-oriented instruments, while consumption subsidies represented only 10.00%, indicating that demand-side policies are overly concentrated on eligibility determination and insufficiently activate direct consumer demand. It is recommended that, building on the existing payment provision framework, targeted consumption subsidies be introduced for low-income disabled families in ethnic and border counties, with subsidy levels differentiated according to household income and disability grade. In government procurement, preference should be given to local small and medium-sized nursing institutions in ethnic areas, cultivating local care service supply capacity through government-purchased services and reducing reliance on a single payment-provision instrument.

#### 4.2.3 Institutionalizing the progressive coverage and consumption voucher coordination model

The policy timeliness (X2) scores of all cities were generally low, with none having established long-term plans (X2-3 = 0 for all). At the autonomous region level, a phased schedule has been planned—“exceeding 5 million enrollees in 2025, exceeding 10 million in 2026, and achieving basic full coverage by the end of 2028” (26)—yet this target has not been incorporated into the policy texts of individual cities. It is recommended that each city incorporate phased coverage targets into its implementation opinions when revising them, elevating progressive coverage from an implementation strategy to an institutional design. In addition, the “older adults care consumption vouchers + LTCI” benefit coordination model that Guangxi has piloted (with a monthly subsidy of up to 800 CNY, allowing 50 and 40% offsets against home-based and institutional care costs, respectively) (26) has thus far been implemented only at the operational level. It is recommended that this model be incorporated into the formal provisions of each city’s policy documents, transforming the coordination mechanism from local experimentation to institutionalized arrangement.

#### 4.2.4 Filling the institutional gaps in full-chain supervision and pluralistic participation

Pre-supervision (X8-1) and post-supervision (X8-3) were absent in multiple cities, with no institutional arrangements established for pre-disability-assessment access review or post-benefit-termination outcome evaluation. It is recommended that a full-chain supervision mechanism covering “application review–process monitoring–outcome evaluation” be established, with clear provisions for disability assessment reconsideration procedures and grievance mechanisms, as well as periodic follow-up and outcome evaluation systems following benefit termination. Social donors were entirely absent from demand-oriented instruments (accounting for only 1.00%), and the encouragement of social donations remains at the level of aspirational expression. It is recommended that the receiving entities for social donations, the scope of fund use, incentive measures for donors, and the modalities for integration with the LTCI fund be clearly specified, so that social force participation can be guided by clear institutional rules.

### 4.3 Limitations and future directions

First, this study only analyzed the content of policy texts and did not conduct a systematic implementation performance evaluation across the five cities. Although we cited some publicly reported implementation data (such as 4.2344 million enrollees and 26,100 benefit recipients), the limited availability of data prevented us from directly linking policy text quality with actual operational outcomes in each city. Liang’s (24) evaluation of the pilot implementation of the LTCI system in City N (Nanning), Guangxi, provides a useful reference: that study found, from the three dimensions of structure, process, and outcomes, that City N faced notable issues including a narrow scope of covered participants, limited coverage, insufficient policy publicity, and low public awareness. These findings corroborate our observations of “demand-side instrument simplification” and “marginalization of beneficiaries,” suggesting a translational gap between policy text completeness and actual implementation effectiveness. Future research could combine field investigations and panel data to conduct comparative analyses between policy text quality and actual implementation performance across the five cities.

Second, the number of policy documents varied across cities, which may affect the comparability of coding frequencies; however, this study employed percentage-based analyses for cross-city comparisons to mitigate this issue.

Third, although this study has verified the robustness of the rankings under different weighting schemes through sensitivity analysis (Table 11), the assignment of weights itself inherently involves subjective choices. Future research could incorporate expert weighting or entropy weighting methods to further validate the stability of the ranking results. As population aging intensifies, Guangxi’s LTCI policies will need to further strengthen sustainability and service adaptability on the basis of maintaining equity.

## Data Availability

The original contributions presented in the study are included in the article/Supplementary material, further inquiries can be directed to the corresponding authors.
