# Supplementary material for: Quantitative evaluation and optimization of long-term care insurance policy texts in pilot cities of Guangxi Zhuang autonomous region
Source: Front Public Health. 2026 Jul 24;14:1853798. doi: 10.3389/fpubh.2026.1853798 (PMC13447056; doi:10.3389/fpubh.2026.1853798)
Supplement: Supplementary file 1 [file Supplementary_file_1.docx]

长期护理保险政策PMC指数评价编码手册

Coding Manual for PMC Index Evaluation of LTCI Policies

I. Manual Overview

1.1 Purpose

This manual is designed for standardized PMC index coding of the pilot policies for the Long-Term Care Insurance (LTCI) in Guangxi. Each coder (or each independent run of the LLM) must strictly follow the judgment criteria specified in this manual to independently assess every secondary variable of each policy, so as to guarantee the transparency, consistency and reproducibility of the coding results.

1.2 Coding Unit

A complete policy document shall serve as one coding unit. After reading the full text thoroughly, coders shall make an overall judgment for each secondary variable.

1.3 Coding Rules

A binary scoring system (1/0) is adopted:

1 = The policy text meets all judging criteria for the variable

0 = The policy text fails to meet the judging criteria for the variable (including complete absence, only vague references, or partial but incomplete compliance)

Core principle: Strict judgment; better to exclude than to include. In case of ambiguity, the principle of "doubt equals zero" shall be followed, and reasons shall be recorded in the remarks column.

1.4 Permission to Refer to Context

Coders may refer to the following content to assist judgment:

(1) The full text of the policy document (search keywords across chapters)

(2) Metadata such as the policy title, issuing authority, and document number (to help determine the policy level and validity)

(3) However, judgments must be based on the main clauses of the text, and inferences cannot be made merely from titles or chapter headings

2. Operationalization Criteria for Primary and Secondary Variables

X1政策性质（Policy Nature）

Variable Definition: To evaluate what types of provisions are included in the wording of policy texts, reflecting the expressive dimension and effectiveness characteristics of policies.

| coding | secondary variable | Judgment Criteria (All the following requirements must be met simultaneously to assign a value of 1) | Keyword / Phrase Prompt | Positive Example (Compliant) | Counterexample (non-compliant) |
| --- | --- | --- | --- | --- | --- |
| X1-1 | 描述 | ①政策包含针对本地老龄化形势或制度背景的实质性描述；②该描述不少于两个连贯句子；③非仅模板化的一句话背景 | 老龄化形势、失能人口规模、照护需求缺口、试点背景 | “贺州市60岁以上老年人口占比已达16.8%，其中失能半失能老人约3.2万人，照护服务需求日益迫切。为积极应对人口老龄化，根据《国家医保局财政部关于扩大长期护理保险制度试点的指导意见》精神……” | “为健全社会保障体系，制定本办法。”（单句，无本地信息） |
| X1-2 | 建议 | ①政策使用非强制性的鼓励、倡导、引导性语言；②同一句子或条款中不含有“应当”“必须”“不得”等强制性情态动词；③若混合出现，以强制性动词为准，归入X1-3 | 鼓励、引导、探索、倡导、支持社会力量、可 | “鼓励商业保险机构开发与长护险相衔接的补充保险产品。” | “鼓励商业保险机构参与，并应当接受监督检查。”（含“应当”，不满足条件②） |
| X1-3 | 引导 | ①政策使用具有规范性约束力的情态动词；②该条款对特定主体施加了作为或不作为的义务 | 应当、必须、不得、严禁、须、要、严格执行 | “参保人员应当按时足额缴纳长期护理保险费。” | “参保人员可自愿选择护理方式。”（无强制性） |
| X1-4 | 支持 | ①政策包含明确的资源承诺（财政投入、基金支持、税收优惠、用地保障等）；②承诺具有可识别的内容，非笼统表述 | 财政补助、纳入预算、税费减免、优先保障用地、基金划拨 | “市财政每年安排不低于500万元的长护险专项补助资金。” | “各级政府应加大对长护险的支持力度。”（无具体内容） |
| X1-5 | 监管 | ①政策明确规定了监督、检查、考核或处罚要求；②监督的执行主体或对象可被识别 | 监督检查、考核评价、责令改正、通报批评、纳入绩效 | “市医保局每季度对定点护理机构进行服务质量考核。” | “加强长护险工作的监督管理。”（无具体规定） |

X2政策时效（Policy Timeliness）

Variable Definition: Evaluate whether the policy has set goals or deadlines for different time spans, which reflects the planning vision of the policy.

| coding | secondary variable | Judgment Criteria (All the following requirements must be met simultaneously to assign a value of 1) | Keyword / Phrase Prompt | Positive Example (Compliant) | Counterexample (non-compliant) |
| --- | --- | --- | --- | --- | --- |
| X2-1 | 短期（≤3年） | ①政策明确列出具体任务或目标需在≤3年内完成；②提供了可验证的截止日期或时间节点 | 20XX年底前、试点期间（若明确为X年）、第一阶段 | “2025年底前完成全市失能人员摸底调查。” | “逐步推进长护险各项工作。”（无时间限定） |
| X2-2 | 中期（3-5年） | ①政策提出了3-5年跨度的制度建设目标或发展规划；②该目标与日常执行任务有明显层次区别 | 2027年、到2028年、十四五期间、制度建设期 | “到2027年，建成覆盖全民、城乡统筹的长护险制度框架。” | “每年组织一次护理人员培训。”（属年度常规工作，非制度目标） |
| X2-3 | 长期（≥5年） | ①政策包含超过5年的发展愿景或战略规划；②或明确提出“制度定型”“长期规划”等远期目标 | 2030年、2035年、长期、远景、制度定型 | “到2035年，形成与社会主义现代化相适应的长期护理保障体系。” | “本办法有效期5年。”（仅属规范性文件的效力期限，非发展目标） |

X3政策受众（Policy Recipient）

Variable Definition: Evaluate whether the policy covers and clearly stipulates the roles or rules of all key participating entities.

| coding | secondary variable | Judgment Criteria (All the following requirements must be met simultaneously to assign a value of 1) | Keyword / Phrase Prompt | Positive Example (Compliant) | Counterexample (non-compliant) |
| --- | --- | --- | --- | --- | --- |
| X3-1 | 行政部门 | ①政策指名道姓地分配了职责、任务或权限给至少一个行政部门；②部门名称是具体的（非泛称“有关部门”） | 市医保局、市财政局、市卫健委、民政局 | “市医疗保障局负责长护险的行政管理、政策制定和监督管理工作。” | “相关部门各司其职。”（未指定具体部门） |
| X3-2 | 护理机构 | ①政策对护理服务机构（养老院、医院护理科、社区护理站等）作出了专门规定；②内容涉及准入条件、服务标准、行为规范或费用结算等 | 定点护理机构、养老机构、医疗机构、护理院 | “定点护理机构应与参保人员签订服务协议，明确服务内容与标准。” | “服务由相关机构提供。”（无专门条款） |
| X3-3 | 商业保险机构 | ①政策明确提及商业保险机构作为经办、承办、委托管理或产品开发的参与方；②非仅背景描述 | 商业保险公司、承办机构、商保公司、委托经办 | “通过政府招标方式委托商业保险机构经办长护险业务。” | （全篇无任何商业保险机构提及） |
| X3-4 | 参保人群 | ①政策清晰界定了参保人员范围（如城镇职工、城乡居民、灵活就业人员）；②并包含参保规则（登记、缴费义务、待遇资格等） | 参保人员、参保职工、灵活就业人员、城乡居民 | “本市行政区域内参加职工基本医疗保险的人员，同步参加长期护理保险。” | “全体市民均可享受长护险服务。”（无参保规则） |

X4政策功能（Policy Function）

Variable definition: Evaluate whether the functions of the policy in standardization, guidance, accountability determination and classified management are complete.

| coding | secondary variable | Judgment Criteria (All the following requirements must be met simultaneously to assign a value of 1) | Keyword / Phrase Prompt | Positive Example (Compliant) | Counterexample (non-compliant) |
| --- | --- | --- | --- | --- | --- |
| X4-1 | 明确标准 | ①政策确立了量化或技术性标准；②该标准有具体的数字、指标、等级或列表 | 评分X分、限额X元、床护比、照护计划、评定表 | “重度失能等级评定采用Barthel指数评定量表，总分≤40分为重度失能。” | “根据失能程度给予相应的护理待遇。”（无量化标准） |
| X4-2 | 规范引导 | ①政策对特定主体的行为方式提出了规范性要求或引导；②对象是人的行为而非物的标准 | 如实申报、签订协议、建立档案、持证上岗 | “参保人员申请享受长护险待遇，应当如实提供病史资料和失能状况证明材料。” | “护理服务质量应当符合相关标准。”（属X4-1） |
| X4-3 | 明确权责 | ①政策逐条或逐段列出了至少两个不同类别主体的具体权利或职责；②非仅笼统宣称 | 负责……；有权……；履行……义务 | “市医保局负责政策制定与监督管理；市财政局负责基金专户管理与财政补助拨付；定点护理机构负责提供符合协议约定的护理服务。” | “各方应切实履行各自职责。”（未列明） |
| X4-4 | 分类监管 | ①政策对不同类别的对象设定了不同的监管措施或管理规则；②类别和措施的对应关系可被识别 | 分类管理、区别对待、针对……另行规定 | “对公立护理机构和民办护理机构实行分类管理。民办机构在市场准入、政府购买服务等方面与公立机构享受同等待遇。” | “加强对各类护理服务主体的监督管理。”（无分类） |

X5政策内容（Policy Content）

Variable Definition: To assess the completeness of the core institutional components of the policy (coverage targets, funds, procedures, administration, accountability and coordination).

| coding | secondary variable | Judgment Criteria (All the following requirements must be met simultaneously to assign a value of 1) | Keyword / Phrase Prompt | Positive Example (Compliant) | Counterexample (non-compliant) |
| --- | --- | --- | --- | --- | --- |
| X5-1 | 保障对象 | ①政策用明确的资格标准界定了待遇享受人群；②该标准包含可操作的条件（如失能等级、持续时长） | 重度失能、持续X个月以上、经评定达到X级 | “经失能等级评定为重度失能，且失能状态持续6个月以上的参保人员，可享受长护险待遇。” | “符合条件的参保人员可享受待遇。”（未说明条件） |
| X5-2 | 统筹模式 | ①政策明确写明了基金的统筹模式；②且描述了该模式下的收支管理基本框架 | 市级统筹、县级统筹、统一管理 | “长护险基金实行市级统筹，全市统一政策、统一标准、统一管理。” | （全篇未提及统筹模式） |
| X5-3 | 职能划分 | ①政策以静态列表形式，说明了不同部门各自负责的领域；②不含“会同”“联合”等互动词（有互动词归X5-8） | 负责、承担、主管 | “医保部门负责长护险政策制定；财政部门负责基金监管；卫健部门负责护理机构行业管理。” | “医保部门会同财政部门制定筹资标准。”（含互动词，归X5-8） |
| X5-4 | 基金管理 | ①政策包含专门针对基金账户和资金管理的条款；②非仅支付标准或待遇描述 | 单独建账、独立核算、专款专用、基金预算、收支管理 | “长护险基金纳入社会保障基金财政专户管理，单独建账，独立核算，专款专用。” | “长护险基金用于支付符合规定的护理费用。”（仅属用途说明） |
| X5-5 | 申办流程 | ①政策分步详细描述了从申办到待遇享受的完整操作路径；②各步骤之间有时间先后和逻辑关联 | 提出申请→受理审核→失能评估→公示→签订协议→享受待遇 | “参保人提出申请后，经办机构应在5个工作日内受理并告知评估安排；评估机构在10个工作日内完成评定并出具报告；评定结果公示7天无异议后，次月起享受待遇。” | “申请程序按相关规定办理。”（无具体路径） |
| X5-6 | 监管运行 | ①政策使用可执行的工具（服务协议范本、绩效考核指标、续约/退出条款）来规范经办机构；②非仅笼统要求 | 协议管理、绩效考核、年度评价、续约条件 | “经办机构应与定点护理机构签订服务协议，协议应包括服务内容、质量要求、结算方式及违约责任。” | “加强对经办机构的管理。”（无具体工具） |
| X5-7 | 责任处理 | ①政策列明了具体的违规情形及对应的处理措施；②措施具有可执行性（警告、罚款、取消资格、追回基金等） | 警告、罚款、取消定点资格、追回基金、解约 | “定点机构发生欺诈骗保行为的，追回违规费用，处2-5倍罚款，并取消定点资格。” | “对违规行为严肃处理。”（无明确后果） |
| X5-8 | 协同管理 | ①政策规定了至少两个部门之间的互动式协作机制；②关键词必须体现“互动” | 会同、联合、协调、信息共享、联席会议、配合 | “市医保局会同市卫健委、民政局建立联合监督检查机制，实行信息共享。” | “各部门加强协作配合。”（无具体机制） |

X6政策领域（Policy Field）

Variable Definition: Evaluate whether the policy broadly covers multiple fields including the economy, social services, technology, politics and institutions.

| coding | secondary variable | Judgment Criteria (All the following requirements must be met simultaneously to assign a value of 1) | Keyword / Phrase Prompt | Positive Example (Compliant) | Counterexample (non-compliant) |
| --- | --- | --- | --- | --- | --- |
| X6-1 | 经济 | ①政策涉及资金筹集、支付、财政补贴、费用分担等经济/财务维度；②有具体机制或比例说明 | 筹资标准、缴费比例、财政补助、支付标准、个人分担 | “长护险基金由个人缴费、单位缴费和财政补助构成。个人缴费每人每年30元。” | “长护险应建立多元筹资渠道。”（无具体经济规定） |
| X6-2 | 社会服务 | ①政策涉及照护服务的递送、组织、质量监控或服务方式；②服务内容具有可识别性 | 居家护理、机构护理、社区照护、服务项目、服务时长 | “根据失能等级，提供居家上门护理或机构专业护理服务。居家护理每月服务不少于20小时。” | “提供适宜的照护服务。”（无具体服务内容） |
| X6-3 | 技术 | ①政策明确要求或描述了信息系统、智能化平台、数据共享等技术工具的应用；②技术工具是政策运行的必要组成 | 信息系统、平台、数据库、智能化、线上申请、电子档案 | “建立全市统一的长护险信息管理系统，实现申请、评估、结算、监管全流程线上运行。” | “鼓励运用信息技术。”（仅属倡导） |
| X6-4 | 政治 | ①政策明显体现了政治层级或制度定位；②如引用中央文件作为统领、描述自身在改革中的层级位置 | 贯彻落实X精神、纳入市委重点工作、改革试点任务 | “为贯彻落实《中共中央国务院关于深化医疗保障制度改革的意见》，将长护险试点纳入本市重点改革任务。” | “根据上级有关文件精神……”（过于模糊） |
| X6-5 | 制度 | ①政策明确援引上位法律法规为自己提供制度锚定；②或明确描述自身与某上位制度的衔接关系 | 根据《……法》、依据X发〔202X〕X号文件、是……制度的组成部分 | “根据《中华人民共和国社会保险法》和《国家医保局财政部关于扩大长期护理保险制度试点的指导意见》制定本办法。” | “健全社会保障制度体系。”（仅属宏观表述，无法律锚定） |

X7政策工具（Policy Instruments）

Variable Definition: Evaluate whether the policy comprehensively adopts the three types of policy instruments, namely supply-oriented, demand-oriented and environment-oriented.

| coding | secondary variable | Judgment Criteria (All the following requirements must be met simultaneously to assign a value of 1) | Keyword / Phrase Prompt | Positive Example (Compliant) | Counterexample (non-compliant) |
| --- | --- | --- | --- | --- | --- |
| X7-1 | 供给型 | ①政策通过直接的资源投入来扩大服务供给；②有具体措施（如人才培养计划、机构建设目标、设施配置要求） | 培养、培训、建设、增设床位、配置设备、开设专业 | “支持职业院校开设护理相关专业，每年培养不少于200名专业护理人才。” | “鼓励各方加大护理服务投入。”（无具体措施） |
| X7-2 | 需求型 | ①政策通过购买、补贴、支付设计等手段直接作用于需求方；②使参保人或受益人能够获取具体服务 | 支付、补贴、购买服务、结算、限额、服务券 | “重度失能人员居家护理费用由基金按每月1500元限额支付。” | “满足失能人员的照护需求。”（无支付或补贴机制） |
| X7-3 | 环境型 | ①政策通过建章立制、规划引导、监管设计来构建运行环境；②间接影响供需而非直接配置资源 | 制定办法、建立机制、规划目标、监管制度、信息共享 | “建立长护险服务质量评价体系和信息公示制度。” | “推动长护险健康发展。”（无制度化手段） |

X8政策监管（Policy Supervision）

Variable Definition: Evaluate whether the policy has formed a full-process regulatory loop covering pre-implementation, ongoing implementation and post-implementation stages.

| coding | secondary variable | Judgment Criteria (All the following requirements must be met simultaneously to assign a value of 1) | Keyword / Phrase Prompt | Positive Example (Compliant) | Counterexample (non-compliant) |
| --- | --- | --- | --- | --- | --- |
| X8-1 | 事前监管 | ①政策在服务发生前设置了准入审核或资格审查机制；②有明确的通过/不通过标准 | 准入审核、资格审批、协议签订、资质审查、定点准入 | “护理机构申请成为定点机构的，市医保局应在20个工作日内完成资质审核，符合条件的方可签订服务协议。” | “机构应具备相应资质。”（无审核程序） |
| X8-2 | 事中监管 | ①政策在服务运行过程中设立了持续性的监测、检查或审计机制；②该机制具有制度化特征 | 日常巡查、专项检查、费用审核、动态监测、年度考核 | “经办机构每季度对定点机构开展不少于一次的服务质量巡查，并出具巡查报告。” | “加强服务质量监督。”（无检查安排） |
| X8-3 | 事后监管 | ①政策在违规行为发生后或年度终了时设立了问责、处理或退出机制；②该机制对既往行为有追溯力 | 处罚、追回、取消资格、退出、黑名单、通报 | “年度考核不合格且整改不到位的，解除服务协议，两年内不得重新申请定点。” | “对违规机构进行相应处理。”（无可执行后果） |

X9激励约束（Incentive and Constraint）

Variable definition: Evaluate whether the policy contains incentive and restrictive measures including participation incentives, legal guarantees, talent training, risk prevention and control, and institutional coordination.

| coding | secondary variable | Judgment Criteria (All the following requirements must be met simultaneously to assign a value of 1) | Keyword / Phrase Prompt | Positive Example (Compliant) | Counterexample (non-compliant) |
| --- | --- | --- | --- | --- | --- |
| X9-1 | 财政补贴 | ①政策提供了具体化或程序化的财税优惠或补贴；②有可识别的对象、方式或标准 | 财政补贴、税收减免、奖励性补助、运营补贴、以奖代补 | “对考核优秀的定点机构，市财政按每床每年1000元给予运营奖励。” | “对积极参与长护险的机构给予适当支持。”（无量化或程序） |
| X9-2 | 法律保障 | ①政策援引了明确的上位法律法规作为制度法律依据；②或载有法律保障条款（如权利救济途径） | 根据《XX法》、依法享有、行政复议、行政诉讼 | “参保人员对失能评定结果有异议的，可依法申请行政复议或提起行政诉讼。” | “保障参保人员合法权益。”（无法律援引或救济路径） |
| X9-3 | 人才培养 | ①政策规定了结构化的人才发展措施；②如职业教育、资格认证、继续教育学分、政府补贴培训等 | 职业资格、持证上岗、培训补贴、继续教育、定向培养 | “护理人员实行职业资格证书制度，取得证书并从业满一年者，可申请培训补贴2000元。” | “加强护理人员队伍建设。”（无具体措施） |
| X9-4 | 风险管理 | ①政策建立了特定的风险控制机制；②针对基金、信息、操作等维度有制度设计 | 风险预警、基金运行分析、内部审计、举报投诉、信息安全 | “建立长护险基金运行分析和风险预警机制，当基金累计结余低于6个月支付水平时启动预警程序。” | “防范基金运行风险。”（无机制设计） |
| X9-5 | 制度衔接 | ①政策以具体操作路径（转介流程、数据互通、联合经办）实现了与相邻制度的衔接；②非仅提及衔接原则 | 转介、衔接、互通、统一经办、一站办理 | “重度失能人员同时符合残疾人护理补贴条件的，由长护险基金和残疾人护理补贴按比例分担，实行一站式结算。” | “做好与相关保障制度的衔接工作。”（无操作路径） |

3. Coding Records and Summary

3.1 Coding Record Template

Fill in the following record form after each policy code is completed:

政策编号：______政策名称：______编码员：______日期：______

| Primary variable | secondary variable | assignment (1/0) | Basis for assignment (quote key sentences from the original text or explain missing information) |
| --- | --- | --- | --- |
| X1Policy Nature | X1-1 Description |  |  |
|  | X1-2 Suggestion |  |  |
|  | X1-3 Guidance |  |  |
|  | X1-4 Support |  |  |
|  | X1-5 Supervision |  |  |
| …… | …… |  |  |
| X9 Incentive | X9-5 Institutional Coordination |  |  |

3.2 Summary Table of Multiple Inputs and Outputs (for the main body of the thesis)

After completing each policy code, compile them into a multiple input-output table (sample format):

| Primary-level variable | Secondary variable | P1 | P2 | P3 | P4 | P5 |
| --- | --- | --- | --- | --- | --- | --- |
| X1 | X1-1 |  |  |  |  |  |
|  | X1-2 |  |  |  |  |  |
| …… | …… |  |  |  |  |  |
| X9 | X9-5 |  |  |  |  |  |
| 各政策PMC总分 |  |  |  |  |  |  |

4. Requirements for reliability test

Independent Scoring: Two coders independently score each secondary variable in accordance with the coding manual.

Reliability Test: Cohen's Kappa coefficient is used to calculate inter-coder reliability, with a required Kappa value of no less than 0.80. The calculation formula is as follows: Kappa=(Po−Pe)/(1−Pe)

where Po denotes the observed agreement rate and Pe denotes the expected agreement rate.

Dispute resolution: For inconsistent entries, the two coders shall discuss and reach a consensus based on the evaluation criteria in the coding manual to generate the final PMC scoring data.

Result reporting: Upon completion of coding, the Kappa value and its corresponding P-value shall be reported to verify that the inter-coder reliability meets the acceptable standard.

5. Boundary Case Library (Calibration Test)

Before formal coding, all coders must independently complete trial coding for the following 10 boundary cases. Only those achieving a consistency rate of no less than 90% with the standard answers can proceed to formal coding.

| Number | Test text | standard answer | Test Points |
| --- | --- | --- | --- |
| T1 | “The municipal medical insurance bureau shall, jointly with the municipal finance bureau, establish and improve an information sharing mechanism for LTCI fund supervision.” | X5-8=1,X5-3=0 | 协同管理vs职能划分（有关键词“会同”“共享”） |
| T2 | “Encourage and guide social capital to invest in the LTCI service sector.” | X1-2=1,X1-4=0 | 建议vs支持（“鼓励”属倡导，无具体资源承诺） |
| T3 | “Designated institutions shall be staffed with at least 5 full-time nursing personnel holding professional qualification certificates.” | X4-1=1,X4-2=1 | 多重功能判定（有量化标准，也有行为规范） |
| T4 | “本办法自2024年1月1日起施行，有效期至2026年12月31日。” | X2-1=0,X2-2=0,X2-3=0 | 时效判定（效力期限≠任务期限或发展目标） |
| T5 | “参保人员按时足额缴纳保险费。” | X1-3=1 | 指引（“应”虽省略但可推断，原文若写“应”则更明确） |
| T6 | “长护险基金支付范围包括：基本生活照料费用、医疗护理费用、康复服务费用。” | X6-1=1,X6-2=1 | 多领域覆盖 |
| T7 | “重度失能人员享受待遇的标准为：居家护理每人每月1200元；机构护理每人每月1800元。” | X7-2=1,X4-1=1 | 需求型工具+量化标准 |
| T8 | “市医保局每年向社会公布长护险基金收支情况。” | X8-3=1 | 事后监管（信息披露属事后公示） |
| T9 | “对以欺诈手段骗取长护险基金的，追回违规费用，并处以骗取金额2倍以上5倍以下罚款。” | X5-7=1 | 问责处理（有具体违规情形和量化处罚） |
| T10 | “根据《中华人民共和国社会保险法》《医疗保障基金使用监督管理条例》制定本办法。” | X6-5=1,X9-2=1 | 制度锚定+法律保障 |

编码员签字：______日期：______一致率：______/10

References

[1]EstradaMR,YapSF,NagarajS.BeyondtheCeterisParibusAssumption:Modeling Demandand Supply Assuming OmniaMobilis[J].SocialScienceElectronicPublishing.2011(33):523-536.
[2]张永安,郄海拓.国务院创新政策量化评价——基于PMC指数模型[J].科技进步与对策,2017,34(17):127-136.
[3]张文静,张丽,姚俊.长期护理保险制度政策评价:基于PMC指数模型[J].中国卫生事业管理,2021,38(02):103-108.
[4]董纪昌,袁铨,尹利君,等.基于PMC指数模型的单项房地产政策量化评价研究——以我国“十三五”以来住房租赁政策为例[J].管理评论,2020,32(05):3-13+75.DOI:10.14120/j.cnki.cn11-5057/f.2020.05.001.
[5]秦亮,张华,吴华珠,等.基于PMC指数模型的数字乡村政策量化评价研究[J].现代管理科学,2023,(01):127-139.
[6]卜令通,张嘉伟.基于PMC指数模型的数字经济政策量化评价[J].统计与决策,2023,39(07):22-27.
