# Supplementary material for: Quantitative evaluation and optimization of long-term care insurance policy texts in pilot cities of Guangxi Zhuang autonomous region
Source: Front Public Health. 2026 Jul 24;14:1853798. doi: 10.3389/fpubh.2026.1853798 (PMC13447056; doi:10.3389/fpubh.2026.1853798)
Supplement: Supplementary file 2 [file Supplementary_file_2.docx]

长期护理保险政策文本政策工具-利益相关者编码手册

Long-term Care Insurance Policy Text: Policy Tools–Stakeholder Coding Manual

1.Overview of the Coding Framework

This coding manual aims to conduct systematic and replicable content analysis on policy documents of Long-term Care Insurance (LTCI). The coding framework consists of two independent dimensions: the Policy Instruments Dimension and the Stakeholders Dimension. The two dimensions follow the mutually exclusive and independent principles during the coding process to guarantee the reliability and validity of the analysis results.

1.1 Theoretical Basis

Based on the policy tool classification theory proposed by Rothwell & Zegveld (1985) and the policy practice of the long-term care insurance system, the policy tool dimension is divided into three major categories: supply-side, demand-side and environmental types, which are further subdivided into 12 subcategories. Based on Freeman's stakeholder theory (1984) and the governance structure of China's long-term care insurance system, eight types of core stakeholders are identified in the stakeholder dimension.

1.2 Coding Unit

Natural sentences serve as the basic analysis unit. Complete items introduced by periods, semicolons and colons are taken as the initial segmentation boundaries.

However, consecutive multiple items shall be combined into one coding unit if any of the following conditions is met:

(1) Multiple sub-items constitute coordinate predicates or objects belonging to the same main clause；

(2) f each item is split separately, the semantics will be incomplete, making it impossible to independently determine the category of policy instruments；

(3) All sub-items serve the same core purpose of the same policy instrument.。

1.3 Context Reference Rules

To ensure coreference resolution and semantic integrity, coders have the right and obligation to refer to the two sentences preceding and following the current sentence to assist in judging the following situations:

1.3.1 Anaphora Resolution

If the current sentence contains anaphoric words such as "the above-mentioned", "the preceding paragraph", "the said" and "its", you must trace back to the referenced content and make a comprehensive judgment combined with the attribute of its policy instrument. The serial number of the referenced sentence shall be marked in the "Basis" column of the coding record.

Example: Original sentence: "Its standards shall be formulated by the Municipal Healthcare Security Administration together with the Finance Bureau." → If the preceding sentence has specified "the payment standard for nursing expenses", the current sentence shall be coded as S-St (standard formulation) with the subject GOV.

1.3.2 hared Subject for Clauses

If the current sentence has no subject (e.g., "Implemented in accordance with..."), trace back to the nearest sentence with a subject to identify the actor of the clause and the core policy instrument.

1.3.3 Reference to chapter context

If a sentence itself does not have independent tool characteristics but is contained within a chapter with a clear theme (such as the chapter "Fund Management"), coders may classify it by referring to the chapter theme and shall note "Dependent on chapter context" in the "Basis" column.

2.Policy Tool Coding System

2.1 Definition of Major Tool Categories

| Main Category | English Name | Definition | Core Functions |
| --- | --- | --- | --- |
| 供给型 | Supply-oriented | 政府直接提供或支持政策运行所需的资源、标准、设施和人力 | 奠定制度运行基础 |
| 需求型 | Demand-oriented | 通过购买服务、补贴、支付设计等方式创造或引导服务需求 | 激活服务市场 |
| 环境型 | Environment-oriented | 建立规则、规划、监管和风险控制机制 | 构建制度框架 |

2.2 Operational Standards for Tool Subcategories

2.2.1 Supply-side tool

| subclass | encoding | operational definition | Keyword prompt | typical example sentences |
| --- | --- | --- | --- | --- |
| 资金供给 | S-F | 规定资金的来源、筹集渠道、缴纳比例、划转方式、财政补助、捐助等资源配置行为 | 划转、缴纳、缴费、补助、筹资、资金来源、财政预算、捐助 | “从医保统筹基金中划入80.5元” |
| 设施建设 | S-I | 要求建立或建设硬件系统、软件平台、信息网络、数据库、监控终端等基础设施 | 信息系统、平台建设、网络管理、数据库、信息系统、监控系统 | “建设全市统一的长护险信息管理系统” |
| 人力开发 | S-H | 涉及护理人员、评估人员等的培训、培养、资格认证、技能提升及相应补贴 | 培训、培养、职业资格、技能证书、专项能力、补贴、队伍建设 | “组织护理人员参加专业技能培训” |
| 标准制定 | S-St | 制定、出台、明确各类规则、目录、流程、技术规范、管理办法等规范性文件 | 制定、出台、明确、规定、办法、细则、标准、流程、清单 | “制定长期护理保险实施细则” |

2.2.2 Demand-oriented tool

| subclass | encoding | operational definition | Keyword prompt | typical example sentences |
| --- | --- | --- | --- | --- |
| 公共采购 | D-P | 基金或财政向服务机构、评估机构支付费用以购买特定服务（护理、评估等） | 支付、结算、购买服务、费用拨付、向...支付 | “长护险基金向定点护理机构支付护理费用” |
| 服务外包 | D-O | 将经办管理业务通过招标、委托、购买等方式交由商业保险机构等第三方承担 | 委托、招标、购买服务、承办、外包、第三方机构 | “通过招标方式委托商业保险公司经办” |
| 消费补贴 | D-S | 对参保人或待遇享受者的护理消费进行定额、限额或按比例的货币补偿 | 定额、限额、补贴、支付标准、床日费、每月标准 | “居家护理每人每月限额1200元” |
| 支付设定 | D-Pm | 规定待遇享受的资格条件、起付线、封顶线、除外责任、待遇中止/终止等规则 | 不予支付、除外、暂停、终止、持续X个月、评定低于X分、不设起付线 | “失能状态持续6个月以上可申请” |

Analysis of Public Procurement (DP) and Consumption Subsidy (DS):

(1) Public Procurement: Funds flow from funds or public finance to service providers (institutions) in exchange for their services.

(2) Consumption Subsidy: Funds flow to the demand side (insured persons/beneficiaries) to reimburse their spending on care services.

(3) Core Criterion for Distinction: The identity of the ultimate recipient of funds. If the recipient is an institution, it falls under DP; if the recipient is an individual, it falls under DS.

(4) Payment Design (DPm): It formulates rules including benefit eligibility, deductibles, payment caps and exclusions. It does not directly execute fund disbursement, but sets preconditions instead.

2.2.3 Environment-type tools

| subclass | encoding | operational definition | Keyword prompt | typical example sentences |
| --- | --- | --- | --- | --- |
| 法规管制 | E-R | 规定各方的强制性义务、准入条件、禁止行为、处罚措施、协议管理要求 | 应当、必须、不得、严禁、准入、退出、处罚、协议管理、签订协议 | “定点机构应当与服务人员签订劳动合同” |
| 战略规划 | E-Sp | 描述制度的总体目标、基本原则、发展方向、产业引导、中长期愿景 | 目标、原则、指导思想、力争、探索、推动、鼓励、促进 | “建立覆盖全民的长期护理保险制度” |
| 协同监管 | E-C | 建立多部门协作、考核、巡查、抽查、公示、动态管理、信息共享等监管机制 | 配合、协同、联合、考核、巡查、公示、动态管理、信息共享 | “医保部门会同民政、卫健部门开展联合检查” |
| 风险管理 | E-Rm | 建立基金安全、风险预警、内部控制、举报投诉、信息披露、欺诈防范等机制 | 风险预警、内部控制、基金安全、专款专用、单独核算、举报投诉、信息披露 | “建立长护险基金运行评估和风险预警机制” |

2.2.3 Environment-type tools

Distinction between Strategic Planning (E-Sp) and Standard Setting (S-St):

(1) Strategic Planning: It describes development directions, objectives, principles and encouraging guidelines, with frequently used words including "strive to", "promote", "boost" and "explore".

(2) Standard Setting: It introduces and clarifies specific operational rules, procedures, checklists and quotas.

(3) Core differentiation: Planning defines "the general direction of work", while standards define "the way to implement work". If a sentence covers both aspects, classification shall be determined by the main verb.

Distinction between Statutory Regulation (E-R) and Coordinated Supervision (E-C):

(1) Statutory Regulation: It lays down mandatory obligations, access requirements, prohibited acts, penalty provisions and other rules for all parties concerned.

(2) Coordinated Supervision: It builds dynamic supervision mechanisms including inter-departmental collaboration, assessment, inspections and information sharing.

(3) Sentences focusing on "establishing coordination mechanisms" or "joint inspections" fall under E-C; sentences that set compliance requirements with expressions such as "shall", "shall not" and "must" fall under E-R.

Distinction between Coordinated Supervision (E-C) and Risk Management (E-Rm):

(1) Coordinated Supervision emphasizes cooperation and supervision procedures among multiple stakeholders.

(2) Risk Management focuses on financial and institutional risks such as fund security, internal control, early warning and fraud prevention.

(3) If a sentence contains explicit risk-related terms such as "risk early warning" or "fund security", it shall be classified as E-Rm on a priority basis.

2.3 Judgment of No Policy Instruments

A sentence shall be judged as containing "no" policy instruments if it meets any of the following conditions:

(1) Pure background statement or elaboration of purposes (e.g., "To cope with population aging...")

(2) Definition and explanation of systems or concepts (e.g., "The long-term care insurance referred to in these Measures means...")

(3) Titles of clauses or chapters (e.g., "Chapter 1 General Provisions")

(4) Metadata such as issuing authority, date and signature

3.Stakeholder Coding System

3.1 Definition of Stakeholder Categories

| Category | coding | Definition | typical subject |
| --- | --- | --- | --- |
| 行政部门 | GOV | 各级政府及其职能部门、经办机构、议事协调机构，承担政策制定、执行、监督职能 | 市人民政府、医保局、财政局、卫健委、民政局、社保经办机构、资格评定委员会 |
| 参保单位 | EMP | 依法为职工缴纳保险费用的用人单位 | 各类企业、机关事业单位 |
| 参保个人 | IND | 履行缴费义务的自然人个体（含在职职工、退休人员、灵活就业人员、失业人员） | 在职职工、退休人员、灵活就业人员、失业人员 |
| 待遇人群 | BEN | 经评估符合条件并实际享受长护险待遇的失能人员 | 重度失能人员、保障对象、失能人员 |
| 护理服务机构 | PRO | 提供护理服务的各类机构 | 定点护理机构、养老机构、医疗机构、居家护理服务机构、社区护理机构 |
| 失能评估机构 | EVA | 受委托开展失能等级评定的第三方机构 | 评估机构、劳动能力鉴定中心、评估公司 |
| 商业保险机构 | INS | 通过招标等方式承接经办服务的商业保险公司 | 商业保险公司、承办机构、商保公司、第三方机构 |
| 社会捐助方 | DON | 提供捐赠的自然人、法人或社会组织 | 企业、社会团体、个人、慈善组织、红十字会 |

3.2 Rules for Stakeholder Identification

3.2.1 Explicit Identification

The names of the aforementioned subjects or their clear aliases appear directly in the sentences (e.g., "designated service institutions" refers to nursing service institutions).。

3.2.2 Exclusive Logical Reference

Subject uniquely determined by context (e.g., "insured persons" refers to individual participants; "benefit recipients" refers to the population entitled to benefits).

3.2.3 Multiple entities

Multiple stakeholders can be identified in one sentence and counted separately.

3.2.4 Handling of Dual Roles

The same entity may assume different roles under different policy contexts, yet its category label remains unchanged. Examples are as follows:

(1) Commercial insurance institutions are coded as INS when undertaking agency administration (D-O) and when subject to supervision (E-R);

(2) Insured individuals are coded as IND when bearing payment obligations (E-R) and when benefiting from treatment (D-Pm).

(3) Stakeholder categories are fixed labels for entity types. Differences in their behaviors are reflected through cross-analysis with policy instruments, and no subcategories need to be created.

4.Coding Procedures and Rules

4.1 Coding Decision-Making Process

| Each natural sentence | | |
| --- | --- | --- |
| Step1: Determine whether it is a policy tool | False | Mark "No Tools" |
|  | True | Select the single most core subclass (mutually exclusive) |
| Step2: Determine whether stakeholders are involved | False | Mark as "no subject" |
|  | True | Mark all involved entities (non-exclusive) |

4.2 Mutual Exclusivity Principle (Policy Instruments)

Each sentence (or merged unit) can only be classified into one subcategory of policy instruments. When multiple instrumental features are present, the unique classification shall be determined according to the following decision tree based on core predicates:

| When multiple tool features appear | Evaluation Rules |
| --- | --- |
| Step1：Identify core predicates | Identify the main verb (core action) of the sentence |
| Step2：Judge the policy objectives | If the goal is to establish rules and regulations → environmental policy  If the goal is to directly invest or provide resources → supply-side policy  If the goal is purchasing, subsidies or demand adjustment → demand-side policy |
| Step3：If the distinction still cannot be made, follow this priority order: Environmental > Supply > Demand | |

4.3 Non-exclusivity Principle (Stakeholders)

Multiple stakeholders can be identified in each sentence.

Any entity that is clearly referred to or regulated by the sentence shall be counted.

4.4 Mutual Exclusion Verification Example

| Original Text | Error Classification (Multiple Choice) | Correct classification (unique) | Reason |
| --- | --- | --- | --- |
| “市医保局制定实施细则并划拨资金” | 标准制定+资金供给 | 标准制定 | 核心动作是“制定”，资金划拨为附带 |
| “应当建立信息系统并进行人员培训” | 设施建设+人力开发 | 设施建设 | 先建设系统再培训人员，以首要动作定类 |
| “鼓励商业保险机构开发产品，并委托其经办” | 战略规划+服务外包 | 服务外包 | 明确的市场化委托行为优先于原则性鼓励 |

5.Measures to Ensure Reliability and Validity

5.1 Coder Reliability

Independent Coding: At least two coders shall independently complete the coding of the same policy text.

Reliability Test: Calculate Cohen's Kappa coefficient, with the requirement that Kappa≥0.80.

Kappa=(Po-Pe)/(1-Pe)

Po stands for the observed agreement rate, and Pe stands for the expected agreement rate.

Dispute Resolution: Inconsistent items shall be adjudicated by a third coder or a consensus shall be reached through group discussion.

5.2 Content Validity

The coding framework is constructed based on the policy instrument theory and stakeholder theory, which is theoretically grounded.

The operational definitions of subcategories are formulated according to the actual keywords in policy texts, ensuring face validity.

Two experts in the field of policy research reviewed the coding framework and verified the comprehensiveness of category coverage.

5.4 Coding Record Template

| field | Explanation | Example |
| --- | --- | --- |
| Sentence Number | File-Chapter-Serial Number | P2-B3.12 |
| Excerpt of the original text | full quotation | "Insured persons... may enjoy long-term care insurance benefits in accordance with regulations" |
| Policy Tool Code | One of the 12 subcategories or "None" | D-Pm(支付设定) |
| Stakeholder Code | Multiple choice, separated by "/" | IND/BEN |
| Brief description of classification basis | Briefly explain the reasons for the judgment | "Set the core eligibility criteria for enjoying benefits" |

5.5 Coding Example

| Number | Original text | Tool Code | Subject Code | basis |
| --- | --- | --- | --- | --- |
| P2-B3.13 | “参保人员...经申请失能等级评估认定的重度失能人员，可按规定享受长护险待遇。” | D-Pm | IND,BEN | 设定待遇资格条件，涉及参保人和待遇人群 |
| P3-D2.3 | “市医疗保障经办机构通过公开招标等形式确定有资质的商业保险机构承办长护险。” | D-O | GOV,INS | 委托经办行为，涉及医保机构和商保公司 |
| P1-B3.10 | “试点阶段，长期护理保险基金筹资标准暂定为每人每年115元。” | S-F | 无 | 资金供给的具体数额，不明确指向某主体 |

5.6 Boundary Case Library and Coder Calibration Test

To ensure all coders interpret the rules consistently, every coder must independently complete trial coding for the following 10 boundary cases before official coding, and calculate the consistency rate against the standard answers. Only those with a consistency rate of 90% or higher can proceed to formal coding.

| Number | Original text | Standard Answer (Tool/Subject) | Key test points |
| --- | --- | --- | --- |
| T1 | “市医保局应会同相关部门建立健全信息共享机制。” | E‑C/GOV | 协同监管vs风险管理 |
| T2 | “鼓励社会力量参与长期护理服务。” | E‑Sp/DON | 战略规划的鼓励性表述 |
| T3 | “护理费用由长护险基金和个人按比例分担。” | D‑S/BEN,GOV?注：基金支付属于消费补贴，主体可为BEN和GOV | 消费补贴判定 |
| T4 | “本办法自发布之日起施行，有效期3年。” | 无工具/无 | 元数据剔除 |
| T5 | “参保人员应按时足额缴纳保险费。” | E‑R/IND | 法规管制下的强制性义务 |
| T6 | “市医保局制定失能等级评定标准。” | S‑St/GOV | 标准制定 |
| T7 | “基金不足时，由市财政给予补助。” | S‑F/GOV | 资金供给 |
| T8 | “为积极应对人口老龄化，探索建立长护险制度。” | E‑Sp/无 | 战略规划，无特定主体 |
| T9 | “定点机构不得以任何理由拒绝符合条件的参保人员。” | E‑R/PRO,BEN | 法规管制+多重主体 |
| T10 | “通过公开招标确定商业保险公司，委托其经办。” | D‑O/GOV,INS | 服务外包判定 |

编码员：______日期：______一致率：______/10

6.Explanation of Statistical Analysis

6.1 Statistical Principles

| Analysis dimension | Statistical unit | mutual exclusivity | denominator of the proportion | Remarks |
| --- | --- | --- | --- | --- |
| 政策工具大类 | 句子（单元） | 互斥 | 有工具句子总数 | “无工具”句子单独统计，不纳入工具比例分母 |
| 政策工具子类 | 句子（单元） | 互斥 | 有工具句子总数 | 同上 |
| 利益相关者 | 句子（单元） | 非互斥 | 总句子数 | “无主体”句子计入分母，出现次数为0 |
| 工具-主体关联 | 句子（单元） | 非互斥 | 各工具子类出现次数 | 仅统计同时有工具且有主体的句子 |

6.2 Common Statistical Indicators

Tool Usage Frequency = (Number of sentences containing a certain tool subclass / Total number of sentences) × 100%

Subject Participation Rate = (Number of sentences involving a certain subject / Total number of sentences) × 100%

Tool-Subject Coupling Degree = (Number of sentences containing both a certain tool and a certain subject / Total occurrences of the tool) × 100%

6.3 Visualization Recommendations

Distribution of policy instruments: Stacked column chart (comparison across three documents)

Distribution of stakeholders: Radar chart or bar chart

Linkages between instruments and actors: Heatmap or chord diagram

Appendix: Coding Quick Reference

| Quick Reference of Policy Tools | |
| --- | --- |
| 供给型(S) | 资金供给(S-F)  设施建设(S-I)  人力开发(S-H)  标准制定(S-St) |
| 需求型(D) | 公共采购(D-P)  服务外包(D-O)  消费补贴(D-S)  支付设定(D-Pm) |
| 环境型(E) | 法规管制(E-R)  战略规划(E-Sp)  协同监管(E-C)  风险管理(E-Rm) |

| Quick Reference for Stakeholders | | | | | |
| --- | --- | --- | --- | --- | --- |
| GOV | 行政部门 | EMP | 参保单位 | IND | 参保个人 |
| BEN | 待遇人群 | PRO | 护理服务机构 | EVA | 失能评估机构 |
| INS | 商业保险机构 | DON | 社会捐助方 |  |  |
